# Results from a Knowledge, Attitudes, and Practices Survey in Two Malaria Transmission Foci of Santo Domingo, Dominican Republic

**DOI:** 10.4269/ajtmh.22-0346

**Published:** 2023-02-27

**Authors:** Hunter Keys, Kevin Bardosh, Keyla Ureña, Luccene Desir, Manuel Tejada, Gregory S. Noland

**Affiliations:** ^1^ The Carter Center, Atlanta, Georgia;; ^2^ Center for One Health Research, School of Public Health, University of Washington, Seattle, Washington;; ^3^ Centro de Prevención y Control de Enfermedades Transmitidas por Vectores y Zoonosis, Santo Domingo, Dominican Republic

## Abstract

Metropolitan Santo Domingo has accounted for a majority of reported malaria cases in the Dominican Republic in recent years. To inform malaria control and elimination efforts, a cross-sectional survey of malaria knowledge, attitudes, and practices collected 489 adult household-level questionnaires across 20 neighborhoods in the city’s two main transmission foci, Los Tres Brazos (*n* = 286) and La Ciénaga (*n* = 203), in December 2020. Overall, most residents (69%) were aware of the problem of malaria in Santo Domingo, but less than half knew that mosquitos transmit the disease (46%) or took any correct preventative measure (45%). More residents of Los Tres Brazos, where malaria incidence is higher than in La Ciénaga, said that they had never been visited by active surveillance teams (80% versus 66%, respectively; *P* = 0.001), did not link mosquitos with malaria transmission (59% versus 48%, *P* = 0.013), and did not know medication can cure malaria (42% versus 27%, *P* = 0.005). Fewer residents of Los Tres Brazos said that malaria was a problem in their neighborhoods (43% versus 49%, *P* = 0.021) and fewer had mosquito bed nets in their homes (42% versus 60%, *P* < 0.001). The majority (75%) of questionnaire respondents in both foci did not have enough mosquito nets for all household residents. These findings demonstrate gaps in malaria knowledge and community-based interventions and highlight the need to improve community engagement for malaria elimination in affected areas of Santo Domingo.

## INTRODUCTION

The island of Hispaniola, shared by the Dominican Republic (population 10.8 million) and Haiti (population 11.6 million), is the only remaining malaria-endemic island in the Caribbean.^1^^,^^2^ Malaria is a potentially fatal parasitic disease that, on Hispaniola, is caused by *Plasmodium falciparum* and transmitted by infected *Anopheles albimanus* mosquitos.^1^ Transmission occurs year-round with seasonal peaks typically observed in May–June and November–December.^3^ Malaria parasites on Hispaniola remain susceptible to chloroquine, which is used in combination with primaquine as first line treatment of uncomplicated malaria.^4^ In 2009, a binational plan targeted malaria elimination in both the Dominican Republic and Haiti by 2020.^5^

**Figure 1.**
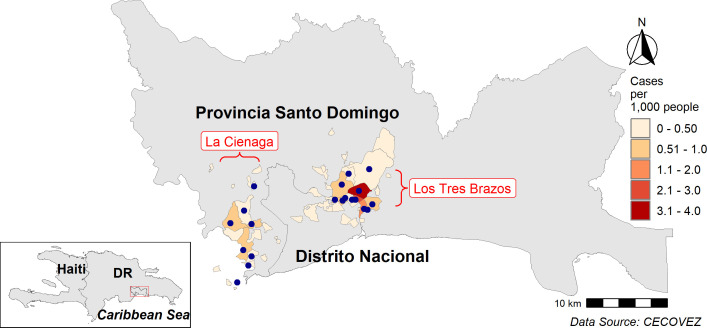
Map of Santo Domingo with La Ciénaga and Los Tres Brazos malaria transmission foci indicated. Neighborhoods shaded according to malaria incidence; blue dots indicate sampled neighborhoods in knowledge, attitudes, and practices survey.

**Figure 2.**
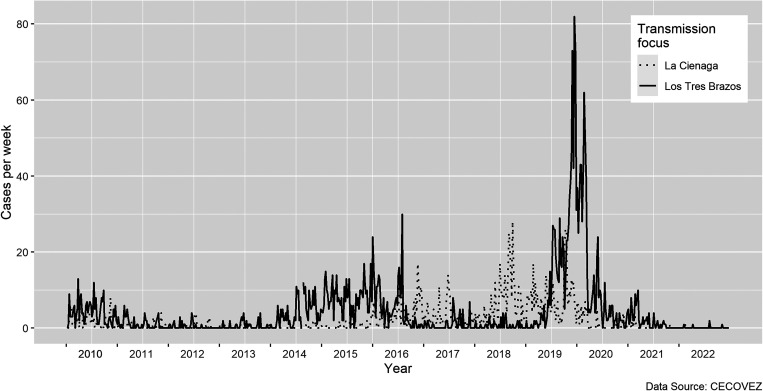
Weekly malaria incidence in two main transmission foci, Santo Domingo, 2010–2021.

Malaria in the Dominican Republic, which reported less than 1,000 cases annually in 9 of the past 10 years, historically has been a rural disease occurring along the border with Haiti and in agricultural areas dependent on migrant labor.^6^ Despite major strides in reducing malaria in rural regions,^7^ transmission began to increase in 2014 in Santo Domingo, the nation’s capital. Since then, the metropolitan area (population 3.3 million) has accounted for the majority of all cases nationwide (average of 75.9% of cases annually between 2015 and 2020). Transmission in the capital region has been concentrated in two main foci: Los Tres Brazos (population 1.3 million), located in the urban core of the city and La Ciénaga (population 482,735), a periurban area on the western fringe of the city (Figure 1). After sustained transmission in both foci in 2010 and early 2011, reported malaria cases fell to less than five cases per week for the remainder of 2011 and throughout 2012 and 2013 (Figure 2). From 2014 to 2016, transmission increased in Los Tres Brazos, with an increase in both the number of passively detected cases and a 2- to 3-fold increase in the proportion of cases detected by active surveillance. As transmission declined in Los Tres Brazos in mid-2016, transmission increased in La Ciénaga through mid-2017 and again from mid-2018 to late 2019, at which time a major resurgence appeared in Los Tres Brazos focus, peaking at 82 cases per week in week 49, 2019. Reasons for this shift in malaria’s epidemiology from rural to urban areas, and the alternating nature of outbreaks between the two foci, remain unknown but are likely linked to rural-to-urban and intraurban migration, favorable vector conditions, population density, and structural issues of poverty and healthcare access in the city.

As cases first increased in Los Tres Brazos in 2014, the national malaria program, part of the country’s vector-borne disease control agency (Spanish acronym: CECOVEZ), was in charge of all field response activities for malaria outbreaks in the country. This vertical orientation entailed deploying CECOVEZ field technicians to malaria-affected neighborhoods (barrios) for fumigation, mosquito-net distributions, and active community surveillance based on door-to-door fever screening, testing of febrile individuals with rapid diagnostic tests for malaria, initiating treatment in the community, and blood slide collection for confirmatory diagnosis by microscopy (following national diagnostic policy). This approach gradually shifted from standard, “top-down” interventions to involving community residents more directly in outbreak control.^8^ This community engagement approach was associated with a reduction in cases in late 2016. However, as the outbreak in Los Tres Brazos subsided in 2016, the Ministry of Health decentralized the country’s malaria program so that CECOVEZ delegated response activities to local-level health districts, which tend to be underresourced and lack malaria expertise. Outbreaks have persisted since decentralization began, including in La Ciénaga focus, characterized by crowded, informal settlements and large bodies of stagnant water. In mid-2019, CECOVEZ began helping local health districts in La Ciénaga scale up networks of trained community residents to undertake active surveillance in their barrios. However, unlike the previous experience in Los Tres Brazos, these residents were also trained to initiate treatment of positive cases diagnosed during active case detection. CECOVEZ relaunched the same strategy in Los Tres Brazos at the end of 2019. Since mid-2019, CECOVEZ and local health districts have recruited and trained 23 trained “community collaborators” (Spanish acronym: COLCOM) in La Ciénaga and 39 in Los Tres Brazos. All malaria response activities in both community and clinical settings continue to follow the advice of the Pan-American Health Organization, which recommends confirmatory diagnosis and initiation of treatment of all suspected cases within the first 48 hours of symptom onset.^9^

In light of the ongoing malaria outbreaks in Santo Domingo, the complexities brought about by the decentralization policy, and recent community-based interventions, this study sought to characterize malaria knowledge, attitudes, and practices (KAP) of residents in both transmission foci. We hypothesized that low levels of these attributes among residents contributes to sustained transmission in these areas. Furthermore, although other studies on Hispaniola focused on malaria KAP in Haiti from both qualitative^10^ and quantitative^11^ perspectives, we are not aware of any published study on malaria KAP in the Dominican Republic. This KAP study seeks to fill that gap by identifying potential program weaknesses and help guide the broader malaria elimination effort on the island.

## MATERIALS AND METHODS

### Study area.

The study area comprised barrios within the two main malaria transmission foci of Santo Domingo (Figure 1). Los Tres Brazos is located in the Santo Domingo Province and the eastern edge of the National District at the confluence of the Ozama and Isabela rivers. Population density is 18,500 persons per km^2^, with residents typically living in older, more organized barrios. La Ciénaga extends across parts of three provinces: Santo Domingo, San Cristobal, and part of the National District. Literally “the swamp” (and not to be confused with the La Ciénaga area along the Ozama River once endemic for lymphatic filariasis^12^), La Ciénaga encompasses a low-lying, poorly drained area once used for agricultural production that has since become the site of massive population growth. In the past decade, thousands of mostly impoverished people from the interior of the country have moved into La Ciénaga in search of work, building homes on once-vacant land. Although statistics are lacking, the focus is considered less densely populated than Los Tres Brazos due to its peripheral location on the outskirts of the city and the preponderance of settlements in semirural areas. Both foci are replete with stagnant canals, thick vegetation, and inadequate sewerage and public works infrastructure.

### Target and sample population, eligibility criteria, and human subjects protection.

The target population was all adults residing in selected high incidence barrios of La Ciénaga and Los Tres Brazos transmission foci. The sample population comprised consenting adult residents of selected households in selected barrios during the survey period.

Participants had to be adults (at least 18 years old), speak and understand Spanish, reside at the selected house, sleep at the selected house every night of the week, and provide oral informed consent. Given that surveying activities mainly took place during the day, there was concern for oversampling housewives (*amas de casa*), the retired and unemployed, and women (who were said to be more likely at home compared with men). Thus, routine, daily checks of the data were done, and feedback was provided to the survey team as needed. As a guiding rule, teams were instructed to alternate sampling men and women.

Participation in the survey was voluntary and uncompensated. No personally identifying information was collected from the participants. Oral informed consent for each participant was obtained after explaining the survey’s purpose, possible risks, and benefits. Consent was then recorded as part of the electronic data file. Refusals were also recorded. The survey protocol was approved by the Dominican Consejo Nacional de Bioética en Salud and the Emory University Institutional Review Board (IRB00099347).

### Survey period.

The study took place from November 30 to December 10, 2020. Surveying was conducted from late mornings to late afternoons. Originally, surveying was planned for late evenings (after 4 pm), when most people were assumed to be home. However, several challenges prevented this: a mandatory evening curfew (part of the national government’s COVID-19 response); logistical difficulty and time to access neighborhoods; and concerns for team safety to stay in neighborhoods after sunset at 6:30 pm.

### Study team.

The study team comprised coinvestigators from The Carter Center and CECOVEZ; two field supervisors from CECOVEZ; and nine Dominican surveyors, six of whom held field positions within CECOVEZ. A 2-day training session reviewed the scope of the survey and its objectives, ethics and informed consent, interviewing techniques and bias, and survey content. The questionnaire was piloted and adjusted before formal surveying activities.

### Survey design and sample overview.

The survey used a single-stage, household-based, cross-sectional design. Rather than generalize findings to the entire population of both foci, the survey purposely selected barrios reporting highest malaria incidence. This decision was taken in the interest of generating a detailed description of KAP within each high-incidence barrio where malaria program activities may need to be tailored. All barrios (*N* = 51) in both foci were ranked according to malaria incidence from January 1 to November 8, 2020. Malaria cases included those reported to CECOVEZ from passive (health facility) and active surveillance. The top 24 barrios were selected with the goal of obtaining 20 questionnaires per barrio (desired sample size: *N* = 480). However, four barrios (two in each focus) had to be eliminated because of safety and logistics concerns. Consequently, 20 total barrios comprised the final sampling frame. Malaria incidence range of the 20 barrios was 0.10 to 3.26 cases per 1,000 persons (median: 0.57 cases per 1,000 persons). The top five barrios were in Los Tres Brazos, including two with exceptionally higher incidence (3.26 per 1,000 persons and 2.99 per 1,000 persons). The total population of these 20 barrios was 704,909. Twelve barrios (population 601,216) were in the Los Tres Brazos focus (overall malaria incidence of the 12 selected barrios, 0.98 per 1,000 persons) and eight barrios (population 103,693) were in the La Ciénaga focus (overall incidence of the eight selected barrios, 0.40 per 1,000 persons). The team increased the number of questionnaires per barrio to 24 to reach the original desired sample size of 480.

**Figure 3.**
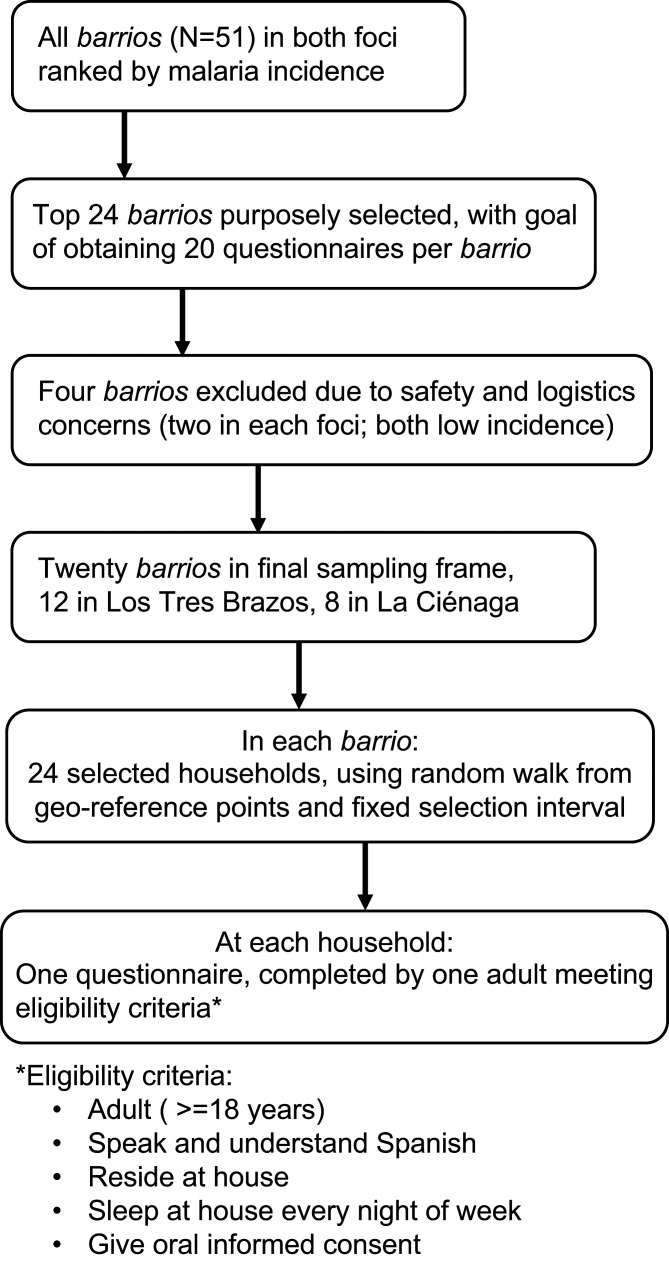
Survey sampling process.

In each barrio, households were selected using the random walk procedure starting from three designated geo-reference points. Study coordinators familiar with the foci used Google Earth and Google Maps to identify four geo-reference points in each barrio that corresponded to a residential (as opposed to agricultural, industrial, or commercial) area. Care was taken to ensure that reference points were spatially distanced throughout the barrio while balancing logistics and safety concerns. One of the four reference points was then randomly eliminated. A surveyor was assigned to each of the remaining three reference points to start data collection after a fixed interval between each completed survey. Figure 3 summarizes the selection process.

### Survey questionnaire.

The survey consisted of seven modules (listed in order): 1) demographics; 2) general health problems in the community, care-seeking practices for recent fever (defined simply as “the last time you had fever”), and last contact with active case detection teams; 3) malaria knowledge and prevention; 4) malaria practices; 5) malaria attitudes; 6) community participation and trust; and 7) socioeconomic status, measured by ownership of household assets. The module on community participation and trust was partially inspired by the USAID Latin American Public Opinion Project 2017 survey; results from that module will be reported separately.

The survey template was created digitally using NEMO software^13^ and then downloaded onto Samsung Galaxy handheld tablets. Survey responses were recorded by surveyors and data were then uploaded to a secure NEMO server.

### Variables and manipulation.

#### Main variables of interest.

Each of the questionnaire’s seven modules contained main variables of interest. *Demographics* captured sex, age, number of residents in the household, time living in the barrio, education, occupation, degree of financial hardship, and whether the participant has health insurance. *General health problems and recent fever practices* captured the participant’s biggest perceived health problem in the barrio, practices regarding last fever (such as first course of action, home remedies, and care source), whether an at-home or facility-based blood test for malaria was ever performed, and whether the participant was diagnosed with malaria. The *malaria knowledge* module asked whether the participant had ever heard of the problem of malaria in the capital, named mosquito bites as a means of transmission, linked stagnant water to malaria, knew cardinal symptoms (e.g., fever, chills), named correct prevention methods, and understood how malaria is diagnosed. This module also inquired about sources of information about malaria. *Malaria attitudes* ascertained whether the participant considered malaria to be a problem in his or her barrio, if most fevers indicated malaria infection, and how motivated the participant felt to take part in antimalaria mass drug administration (MDA) despite perceived risks of COVID. *Malaria practices* asked the participant about the first course of action if sick with malaria symptoms, whether the participant does any correct prevention method around the home, whether the household has enough mosquito nets for all residents, whether the participant has a mosquito net for herself/himself, whether he or she slept under the net the previous night, and if the participant had ever seen indoor residual spraying (IRS) activities in the barrio and whether he or she allowed IRS in the selected home.

Participants could provide multiple responses to some variables, so new, binary variables were created in those instances. For ease of interpretation and analysis, binary variables were created if the participant named mosquito bites or stagnant water as sources of transmission; if the participant named fever or chills as malaria symptoms; if the participant did not know any symptom of malaria; if the participant named any correct prevention method; if the participant took any correct action to eliminate stagnant water around the home; and whether the participant named any of the given sources of information about malaria (friends/family; *promotora* (health promoter) or COLCOM; clinic doctor; educative talks, or *charlas*; digital social networks; radio; and TV).

#### Creation of wealth indices.

Characterization of socioeconomic status was based on principal components analysis (PCA) of household assets and housing materials. Nine variables (assets) ranging from 5% to 95% ownership were included. A continuous wealth score variable based on ownership weight of each asset was created. A cross-tab analysis was done and confirmed that “own computer,” “has Internet,” “owns car,” “has concrete roof,” and “has ceramic tiled floor” were associated with rising wealth. The first component from this second round of PCA explained 46.2% of the variance. Post-PCA testing of item intercorrelation and adequate sampling were done using Bartlett’s test of sphericity and the Kaiser–Meyer–Olkin (KMO) test. Each were satisfactory (Bartlett’s test *P* < 0.01; KMO = 0.72). This linear wealth score was then converted into quintiles and again cross-checked with ownership of assets. A consistent, positive association with rising wealth quintile was noted, so this wealth index variable was ultimately used in subsequent analyses.

### Data analysis.

All data were downloaded from NEMO and then aggregated into an Excel spreadsheet to check for clarity and data mismatch errors. Stata statistical software was used for analysis.^14^ No weighting adjustments were made to the data because the study did not seek population-level estimates. Crude frequencies and proportions were calculated for residents in both transmission foci and for the overall sample. Significant differences between the two foci were detected for categorical and continuous variables using χ^2^ and *t* tests, respectively, although Fisher’s exact test was used for tabulations in which there were five or fewer counts in any given cell. To improve statistical robustness, responses coded as missing or “Don’t Know” were excluded from the analysis if cell counts were less than five. A χ^2^ test-for-trend was done for ordinal variables (education level, wealth quintile, degree of financial hardship, motivation to participate in MDA). Significance was defined as *P* < 0.05.

## RESULTS

A total of 503 survey questionnaires were collected. Of them, 489 questionnaires were completed by eligible, consenting adults. Fourteen (14) eligible individuals refused participation (response rate = 97%). Twelve (12) neighborhoods were sampled within the Los Tres Brazos focus (*N* = 286) and eight were in the La Ciénaga focus (*N* = 203) (Table 1).

**Table 1:** Overview of study area, Santo Domingo, 2020

Transmission focus	Population	Malaria incidence January 1–November 15, 2020 (cases per 1,000 residents)	Total trained community collaborators* mid-2019–December 2020	Sampled barrios	Completed individual questionnaires
Los Tres Brazos	601,216	0.98	39	12	286
La Ciénaga	103,693	0.40	23	8	203
Total	704,909	0.89	62	20	489

* COLCOM: These individuals are residents of their barrios and trained to undertake active case detection and initiate treatment at point-of-diagnosis using rapid diagnostic tests.

### Demographics.

Table 2 shares findings from the demographic portion of the questionnaire. The sample was evenly divided between men (49.7%) and women (50.3%). Average age was 46.9 years (range: 18–90). On average, roughly four people lived in each sampled household (range: 1–15). No significant differences between the two foci were found regarding sex, age, or number of residents per household. However, on average, participants in Los Tres Brazos tended to reside longer in their respective neighborhoods compared with participants in La Ciénaga (24.7 versus 18.1 years, respectively; *P* < 0.001).

**Table 2:** Demographic characteristics of survey participants, by transmission focus, Santo Domingo, 2020

Variable	La Ciénaga (*n* = 203)	Los Tres Brazos (*n* = 286)	Total (*N* = 489)	*P*
Sex, *n* (%)				
Male	100 (49.3)	143 (50.0)	243 (49.7)	0.872
Female	103 (50.7)	143 (50.0)	246 (50.3)	
Age, mean (range), SD	46.7 (18–88), 18.0	47.0 (18–90), 16.6	46.9 (18–90), 17.2	0.843
No. of persons in household, mean (range), SD	3.9 (1–15), 2.0	3.7 (1–10), 1.8	3.8 (1–15), 1.9	0.149
No. of years residing in neighborhood, mean (range), SD	18.1 (1–81), 15.2	24.7 (1–64), 15.8	22 (1–81), 15.9	**< 0.001**
Highest level of education, *n* (%)*				
Completed secondary or more	93 (46.0)	98 (34.3)	191 (39.1)	**0.031**
Completed primary or some secondary	55 (27.2)	97 (33.9)	152 (31.2)	
Some primary or none	54 (26.7)	91 (31.8)	145 (29.7)	
Occupation status, *n* (%)*				
Unemployed	43 (21.2)	30 (10.5)	73 (15.0)	**0.011**
Informal work, homemaker, or student	53 (26.1)	80 (28.1)	133 (27.3)	
Employed	93 (45.8)	157 (55.1)	250 (51.2)	
Retired	14 (6.9)	18 (6.3)	32 (6.6)	
SES quintile, *n* (%)*				
First (richest)	50 (24.8)	47 (16.6)	97 (20.0)	**0.019**
Second	44 (21.8)	53 (18.7)	97 (20.0)	
Third	25 (12.4)	46 (16.2)	71 (14.6)	
Fourth	39 (19.3)	61 (21.5)	100 (20.6)	
Fifth (poorest)	44 (21.8)	77 (27.1)	121 (24.9)	
Financial stress, *n* (%)*				
Monthly income is good enough, and you can save from it	5 (2.5)	11 (3.9)	16 (3.3)	0.423
Monthly income is just good enough, and you do not have major problems	24 (11.9)	37 (13.0)	61 (12.6)	
Monthly income is not good enough, and you are stretched	85 (42.3)	121 (42.6)	206 (42.5)	
Monthly income is not good enough, and you are having a hard time	87 (43.3)	115 (40.5)	202 (41.7)	
Has health insurance, *n* (%)				
Yes	158 (77.8)	235 (82.2)	393 (80.4)	0.234
No	45 (22.2)	51 (17.8)	96 (19.6)	

SES = socioeconomic status.

* Excludes missing responses and/or those who answered “Don’t know” unless explicitly listed. Bold values indicate *P* < 0.05.

Significant differences in education were noted between the two foci (*P* = 0.031), with more people having completed secondary education in La Ciénaga (46%) compared with those in Los Tres Brazos (34.3%). Significant differences (*P* = 0.011) were also noted in proportions of occupation type between the two foci. For example, proportionally more participants in La Ciénaga were unemployed (21.2%) compared with Los Tres Brazos (10.5%), whereas proportionally more people in Los Tres Brazos had formal employment (55.1%) compared with La Ciénaga (45.8%). Along with these differences in education and occupational status, there was also a significant difference in wealth status between the two foci. In general, people in higher socioeconomic quintiles resided in La Ciénaga compared with Los Tres Brazos (*P* = 0.019). However, there was no significant difference in self-reported financial hardship—that is to say, the hardship of poverty appeared to affect the lives of participants in both foci in similar ways. Despite their economic precariousness, the majority of the sample (80.4%) had some form of health insurance. Most of those with health insurance were enrolled in the country’s publicly subsidized option, SENASA.

### General health problems, recent fever, and contact with active case detection teams.

**Figure 4.**
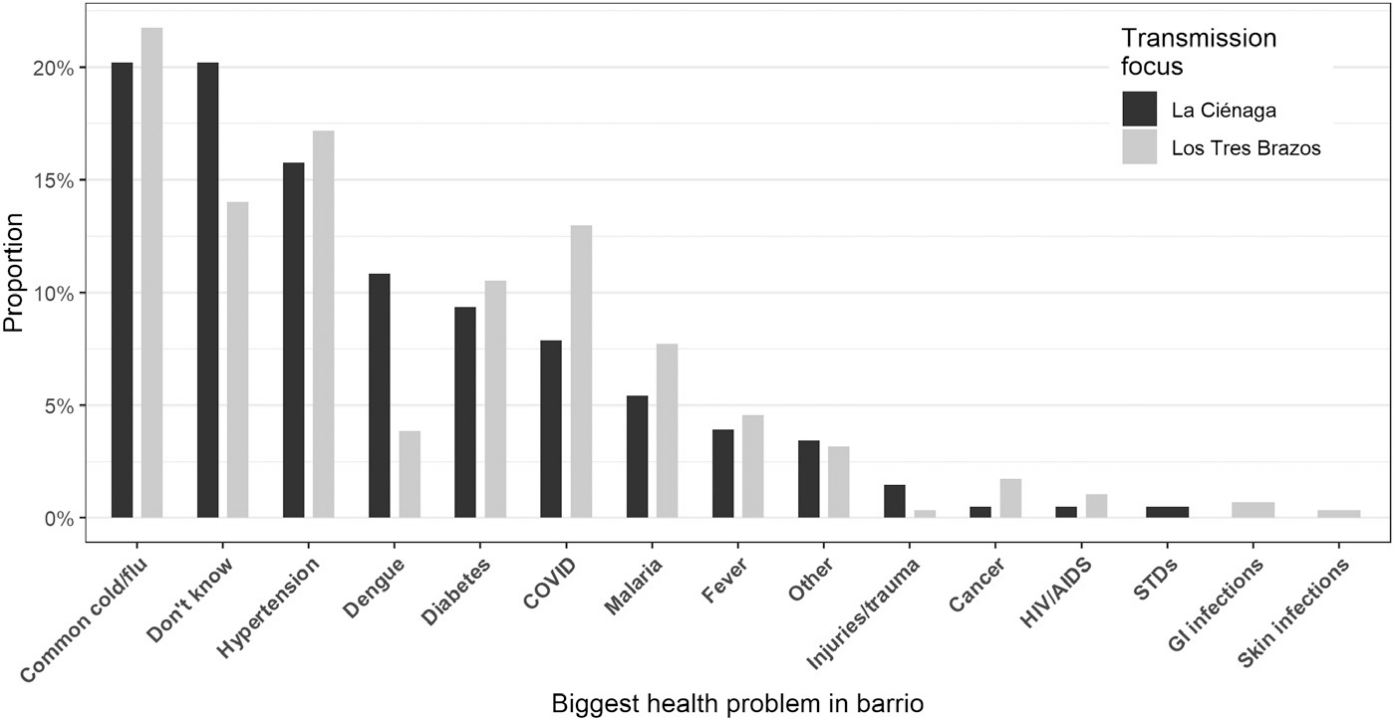
Biggest health problem in barrio, by transmission focus, Santo Domingo, 2020.

In terms of general health problems in barrios, only 6.8% of the sample cited malaria. More common illnesses were the common cold (21.1%), hypertension (16.6%), COVID-19 (10.8%), and diabetes (10%). No significant difference was found among proportions of commonly cited diseases between the two foci (Figure 4).

Table 3 shows results from the recent fever module. Nearly half the sample (42.8%) could not recall or did not know what they did when last ill with fever. Among those who could recall, there was a general preference to self-medicate at home or otherwise wait to see if the condition improved. Just 16.5% of the sample immediately sought care at a clinic or hospital and only one individual reported calling or visiting the local community health worker (CHW). Few participants (2.1%) cited cost as a primary barrier to seeking immediate care. Proportions were not significantly different between the two foci.

**Table 3:** Recent fever practices and contact with active surveillance teams, by transmission focus, Santo Domingo, 2020

Variable	*n* (%)			*P*
	La Ciénaga (*n* = 203)	Los Tres Brazos (*n* = 286)	Total (*N* = 489)	
[For last fever]: What did you do first?*				
Took home remedy	64 (31.7)	113 (39.8)	177 (36.4)	0.342
Went immediately to clinic or hospital	38 (18.8)	42 (14.8)	80 (16.5)	
Waited to see if I get better	9 (4.5)	11 (3.9)	20 (4.1)	
Called CHW	0	1 (0.4)	1 (0.2)	
Don’t know/don’t remember	91 (45.5)	117 (41.2)	208 (42.8)	
Total	202 (100)	284 (100)	486 (100)	
[For last fever, if waited at home or took home remedy]: Why did you wait at home with fever?*				
To see if I get better	30 (41.1)	51 (41.8)	81 (41.5)	0.813
The illness was not serious	24 (32.9)	39 (32.0)	63 (32.3)	
I could not miss work	2 (2.7)	5 (4.1)	7 (3.6)	
It was too expensive to see the doctor	2 (2.7)	2 (1.6)	4 (2.1)	
I do not like the doctors, hospital, clinic	1 (1.4)	6 (4.9)	7 (3.6)	
Other	14 (19.2)	19 (15.6)	33 (16.9)	
Total	73 (100)	122 (100)	195 (100)	
[For last fever, if waited at home or took home remedy]: Eventually, did you seek care?				
Yes	22 (30.1)	25 (20.2)	47 (23.9)	0.113
No	51 (69.9)	99 (79.8)	150 (76.1)	
Total	73 (100)	124 (100)	197 (100)	
[If ultimately sought care for last fever after waiting at home or took home remedy]: Where did you seek care?				
Hospital	13 (59.1)	17 (68.0)	30 (63.8)	0.461
Primary care clinic	5 (22.7)	4 (16.0)	9 (19.2)	
House of CHW	2 (9.1)	0	2 (4.3)	
Other	2 (9.1)	4 (16.0)	6 (12.8)	
Total	22 (100)	25 (100)	47 (100)	
[For last fever, if waited at home or took home remedy, but ultimately sought care]: How long did you wait at home before seeking care?*				
A few days	17 (81.0)	18 (72.0)	35 (76.1)	0.436
More than 1 week	2 (9.5)	6 (24.0)	8 (17.4	
Other	2 (9.5)	1 (4.0)	3 (6.5)	
Total	21 (100)	25 (100)	46 (100)	
If blood test performed for fever (at any care source)*				
Yes	47 (79.7)	44 (64.7)	91 (71.7)	0.062
No	12 (20.3)	24 (35.3)	36 (28.4)	
Total	59 (100)	68 (100)	127 (100)	
Has anyone ever visited your house to do a blood test?*				
Yes	67 (33.7)	57 (20.4)	124 (25.9)	**0.001**
No	132 (66.3)	222 (79.6)	354 (74.1)	
Total	199 (100)	279 (100)	478 (100)	
[If had blood test done at home]: Accept blood test?				
Yes	60 (89.6)	45 (79.0)	105 (84.7)	0.102
No	7 (10.5)	12 (21.1)	19 (15.3)	
Total	67 (100)	57 (100)	124 (100)	
[If had blood test done at home]: When?*				
Within the past month	6 (9.2)	6 (11.3)	12 (10.2)	0.709
More than a month ago	59 (90.8)	47 (88.7)	106 (89.8)	
Total	65 (100)	53 (100)	118 (100)	
Ever been diagnosed with malaria at any time in past				
Yes (includes if last fever was malaria)	10 (5.0)	11 (3.9)	21 (4.3)	0.570
No	192 (95.1)	272 (96.1)	464 (95.7)	
Total	202 (100)	283 (100)	485 (100)	
[If ever diagnosed with malaria]: By whom?*				
CHW	5 (50.0)	1 (11.1)	6 (31.6)	0.269
House visit by public health	1 (10.0)	2 (22.2)	3 (15.8)	
Doctor	4 (40.0)	6 (66.7)	10 (52.6)	
Total	10 (100)	9 (100)	19 (100)	

CHW = community health worker.

* Excludes missing responses and/or those who answered “Don’t know” unless explicitly listed.

The most common points of care for fever were hospitals (63.8%) or primary care clinics (19.2%). Only two participants eventually sought care from a CHW. Most participants who delayed but eventually sought care had waited “A few days” before doing so (76.1%). Among those who visited a care center either immediately or after a delay, 71.7% reported getting a blood test for malaria done at the facility. Thirteen of the 128 (10.2%) individuals who either immediately or eventually sought care were diagnosed with malaria. No significant differences were found between the two foci regarding care preferences for fever or whether a blood test was done for fever at any care source.

The majority of participants in both foci reported not ever being visited at home for a malaria blood test (74.1%). However, proportionally more people in La Ciénaga reported contact with active surveillance teams compared with participants in Los Tres Brazos (33.7% versus 20.4%; *P* = 0.001). The majority of those who had been contacted by an active surveillance team accepted the at-home blood test (84.7%). No significant difference in proportions for accepting at-home testing was noted between the two foci.

Lastly, 4.3% of participants said that they had ever been diagnosed with malaria at any time in the past (including the 13 participants diagnosed during their most recent fever episode), with no significant difference between the two foci. Of those who were diagnosed, almost half (52.6%) were diagnosed by medical doctors, with CHWs the second most common source (31.6%).

### Malaria knowledge.

Table 4 displays results from the malaria knowledge module of the questionnaire. Overall, 69.4% said that they had heard of the problem of malaria in the capital, with nearly equal proportions in both foci (no significant difference). Just under half of the sample (45.6%) said that mosquito bites transmit malaria, although more participants in La Ciénaga said so than in Los Tres Brazos (52.2% versus 40.9%; *P* = 0.013). Most participants (59.7%) named fever and/or chills as symptoms of malaria, whereas 37% could not name any correct symptom. No significant difference in knowledge of malaria symptoms were found between the two foci.

**Table 4:** Malaria knowledge among survey participants, by transmission focus, Santo Domingo, 2020

Variable	*n* (%)			*P*
	La Ciénaga (*n* = 203)	Los Tres Brazos (*n* = 286)	Total (*N* = 489)	
Ever heard of problem of malaria in capital*				
Yes	138 (68.3)	200 (70.2)	338 (69.4)	0.832
No	55 (27.2)	75 (26.3)	130 (26.7)	
Don’t know	9 (4.5)	10 (3.5)	19 (3.9)	
Named mosquito bites as way to get malaria				
Yes	106 (52.2)	117 (40.9)	223 (45.6)	**0.013**
No	97 (47.8)	169 (59.1)	266 (54.4)	
Is there a relationship between stagnant water and malaria?*				
Yes	176 (87.6)	230 (81.3)	406 (83.9)	0.106
No	9 (4.5)	26 (9.2)	35 (7.2)	
Don’t know	16 (8.0)	27 (9.4)	43 (8.9)	
Named fever or chills as malaria symptoms				
Yes	129 (63.6)	163 (57.0)	292 (59.7)	0.145
No	74 (36.5)	123 (43.0)	197 (40.3)	
Don’t know any symptom of malaria				
Yes	69 (34.0)	112 (39.2)	181 (37.0)	0.243
No (able to name any symptom)	134 (66.0)	174 (60.8)	308 (63.0)	
Named any correct prevention method†				
Yes	97 (47.8)	121 (42.3)	218 (44.6)	0.230
No	106 (52.2)	165 (57.7)	271 (55.4)	
[If know how malaria is diagnosed]: How?*				
Blood test	85 (93.4)	79 (91.9)	164 (92.7)	0.902
Other	5 (5.5)	5 (5.8)	10 (5.7)	
Don’t know	1 (1.1)	2 (2.3)	3 (1.7)	
Can medicine cure malaria?*				
Yes	129 (63.9)	140 (49.3)	269 (55.4)	**0.005**
No	18 (8.9)	25 (8.8)	43 (8.9)	
Don’t know	55 (27.2)	119 (41.9)	174 (35.8)	

* Excludes missing responses and/or those who answered “Don’t know” unless explicitly listed.

† Malaria prevention methods could include sleep under bed net, avoid mosquito bites, indoor residual spraying, use repellant, and/or fill in holes/puddles.

Most participants (83.9%) said that there was a relationship between stagnant water and malaria, although responses may reflect some desirability bias. Just under half the sample (44.6%) named any correct method for preventing malaria, such as sleeping under mosquito bed nets, fumigation, using insect repellent, and/or filling in puddles and eliminating stagnant water around the home. Proportions of responses to malaria prevention methods were not significantly different between residents of the two foci.

Among the 36.4% of participants who said that they knew how malaria is diagnosed, 92.7% correctly said diagnosis is done by blood test, with no significant difference between the foci. When asked the question, “Can medicine cure malaria?”, proportionally more people in La Ciénaga said “Yes” compared with people in Los Tres Brazos (63.9% versus 49.3%; *P* = 0.005).

**Figure 5.**
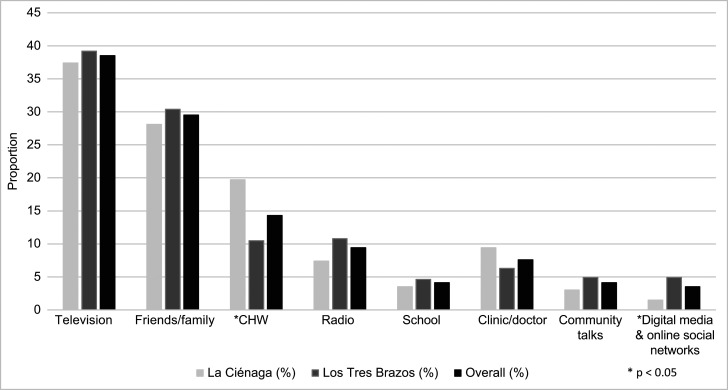
Proportions of survey participants who cited given source of information about malaria, by transmission focus and overall sample, Santo Domingo, 2020.

Figure 5 displays reported sources of information about malaria. Participants tended to say that they learned about malaria via television (38.5%) or through their friends and family (29.5%). To note, 84.9% of the sample owned a television. Only 14.3% named CHWs, with more participants in La Ciénaga (19.7%) naming CHWs than in Los Tres Brazos (10.5%). Only 7.6% of the sample cited clinics or doctors as sources of information for malaria.

### Malaria attitudes and practices.

Table 5 shows results from questions pertaining to malaria attitudes and practices. Just under half (45.3%) of the sample said that malaria is a problem in their barrios. Interestingly, more participants in La Ciénaga (48.5%) said malaria was a problem compared with participants in the Los Tres Brazos focus (43%), where incidence was higher. Proportionally more people in Los Tres Brazos answered “Don’t know” (26.4%) compared with La Ciénaga (15.8%), which likely explains the significant difference (*P* = 0.021) with respect to this variable: there was no significant difference once the analysis of this variable excluded these “Don’t know” responses (*P* = 0.887).

**Table 5:** Malaria attitudes and practices among survey participants, by transmission focus, Santo Domingo, 2020

Variable	*n* (%)			*P*
	La Ciénaga (*n* = 203)	Los Tres Brazos (*n* = 286)	Total (*N* = 489)	
Malaria is a problem in your neighborhood*				
Yes	98 (48.5)	122 (43.0)	220 (45.3)	**0.021** †
No	72 (35.6)	87 (30.6)	159 (32.7)	
Don’t know	32 (15.8)	75 (26.4)	107 (22.0)	
Most fevers are malaria*				
Yes	14 (6.9)	36 (12.7)	50 (10.3)	0.058
No	153 (75.7)	190 (66.9)	343 (70.6)	
Don’t know	35 (17.3)	58 (20.4)	93 (19.1)	
Given COVID, how motivated to participate in antimalaria MDA?*				
Very motivated	110 (56.4)	148 (54.1)	258 (55.1)	0.415
Motivated	58 (29.7)	76 (27.8)	134 (28.6)	
Neutral	13 (6.7)	21 (7.7)	34 (7.3)	
Unmotivated	9 (4.6)	12 (4.4)	21 (4.5)	
Very unmotivated	5 (2.6)	16 (5.9)	21 (4.5)	
If have symptoms, first action*				
Go to hospital/clinic	134 (66.3)	172 (60.6)	306 (63.0)	0.176
Wait to see if I get better	9 (4.5)	8 (2.8)	17 (3.5)	
Call CHW	8 (4.0)	6 (2.1)	14 (2.9)	
Other	11 (5.5)	19 (6.7)	30 (6.2)	
Don’t know	40 (19.8)	79 (27.8)	119 (24.5)	
Does any correct action for stagnant water around home‡				
Yes	146 (71.9)	189 (66.1)	335 (68.5)	0.171
No	57 (28.1)	97 (33.9)	154 (31.5)	
Has mosquito nets in home*				
Yes	122 (60.4)	117 (41.5)	239 (49.4)	**< 0.001**
No	80 (39.6)	165 (58.5)	245 (50.6)	
[If has nets in home]: Has enough mosquito nets for all residents in home				
Yes	30 (24.6)	30 (25.6)	60 (25.1)	0.851
No	92 (75.4)	87 (74.4)	179 (74.9)	
[If has nets in home]: Has mosquito net for self*				
Yes	105 (86.8)	101 (86.3)	206 (86.6)	0.919
No	16 (13.2)	16 (13.7)	32 (13.5)	
[If has net for self]: Slept under net last night				
Yes	87 (82.9)	77 (76.2)	164 (79.6)	0.238
No	18 (17.1)	24 (23.8)	42 (20.4)	
Ever seen IRS in barrio*				
Yes	85 (42.7)	177 (63.2)	262 (54.7)	**< 0.001**
No	114 (57.3)	103 (36.8)	217 (45.3)	
[If saw IRS in barrio]: Allowed IRS at home*				
Yes	74 (89.2)	148 (85.1)	222 (86.4)	0.370
No	9 (10.8)	26 (14.9)	35 (13.6)	

CHW = community health worker; IRS = indoor residual spraying; MDA = mass drug administration.

* Excludes missing responses and/or those who answered “Don’t know” unless explicitly listed.

† After excluding “Don’t know” responses, *P* = 0.887.

‡ Corrective actions for standing water included fill in holes, cover containers, remove tires, put chlorine in standing water.

The majority (70.6%) said that most fevers are not malaria. After explaining the concept of MDA for malaria, the vast majority of the sample said that they were either very motivated (55.1%) or motivated (28.6%) to participate in a MDA campaign despite COVID-19. No significant differences in proportions to these latter two questions were found between the two foci.

Although participants did not generally name clinics and doctors as sources of information about malaria, most participants (63%) still said that they would go to a hospital if sick with symptoms (no significant difference between the two foci). Overall, 68.5% undertook a correct action to eliminate stagnant water around the home, such as filling in puddles or covering standing water (no significant difference between the two foci). Just under half (49.4%) of all participants said that they had mosquito nets in the home, but the majority (74.9%) did not have enough nets for all household residents. More participants in La Ciénaga said that they have mosquito nets in their homes compared with participants in Los Tres Brazos (60.4% versus 41.5%; *P* < 0.001). The majority of those who had a net for themselves said that they slept under the net the previous night (79.6%). No differences were noted in participant bed net use between the two foci.

More participants in Los Tres Brazos than in La Ciénaga (63.2% versus 42.7%; *P* < 0.001) reported seeing IRS teams in their neighborhood. Among those who had ever seen IRS teams in their barrio, the majority (86.4%) allowed teams into their homes.

## DISCUSSION

This survey found a mixed picture of people’s knowledge, attitudes, and practices for malaria in the two principal transmission foci of urban Santo Domingo. Although most residents (69.4%) were aware that malaria is a problem in the capital, less than half (45.6%) said that mosquitos transmit the parasite and just over a third (36.4%) could say how malaria was diagnosed. Despite these apparent gaps in knowledge of malaria’s biology, people still take important steps to reduce malaria transmission. Although just under half the sample (49.4%) had mosquitos nets in the home, the majority of those with nets (79.6%) said that they slept under them. A majority of the sample also took steps to eliminate stagnant water around the home (68.5%) and accepted malaria interventions such as IRS (86.4%) and at-home blood testing when contacted by active case detection teams (84.7%). A small number of participants reported past malaria infection (4.3%). Most expressed motivation to participate in antimalaria MDA (55.1% were “very motivated” and 28.6% were “motivated”), a potentially useful strategy in this area.^15^^,^^16^

There were, however, significant differences between the two foci, suggesting greater gaps in Los Tres Brazos, which is surprising given the longer history of the country’s malaria program in the focus and recent wave of malaria transmission (Figure 2). More participants in Los Tres Brazos than in La Ciénaga said that they had never been visited by an active surveillance team (79.6% versus 66.3%, respectively; *P* = 0.001), did not link mosquitos with malaria transmission (59.1% versus 47.8%; *P* = 0.013), and did not know medication can cure malaria infection (41.9% versus 27.2%; *P* = 0.005). Furthermore, fewer participants in Los Tres Brazos said that malaria was a problem in their neighborhoods (43% versus 48.5%), and fewer had mosquito bed nets in their homes (41.5% versus 60.4%; *P* < 0.001). Other notable, although not significant, differences between the two foci were that proportionally more people in Los Tres Brazos did not ultimately seek care for fever after waiting at home (79.8% versus 69.9%); did not receive a blood test at any care source if they did seek care (35.3% versus 20.3%); refused at-home blood testing by active case detection teams (21.1% versus 10.5%); and did not link stagnant water to malaria transmission (9.2% versus 4.5%). Finally, there was lower exposure to CHWs for malaria information in Los Tres Brazos compared with La Ciénaga (10.5% versus 19.7%; *P* < 0.05).

Although population-level inferences cannot be made given the design of the survey, these findings still suggest a lower level of community-based outreach and malaria KAP in Los Tres Brazos. One potential reason for these findings is due to the ongoing process of decentralizing the country’s malaria program to generally less experienced and underresourced health districts. As described in detail elsewhere,^8^^,^^17^ until 2015–2016, the country’s national malaria program was a vertical structure that entailed sending field teams directly to communities during outbreaks to mobilize community members and train some in active case detection. Since 2016, the malaria program has undergone decentralization to local health districts and assumed an advisory, rather than operational, role. These structural changes have generated some confusion about roles and responsibilities as well as challenges to transferring not only technical competencies (such as blood slide collection) but also the skills in cultivating and sustaining the “human infrastructure” necessary for community-based outbreak control.^17^^,^^18^

When sick with fever, over a third of the sample (36.4%) took a home remedy or waited to see if they improved on their own (4.1%) rather than immediately seek care or alert a nearby CHW (16.7%). Among those who either took a home remedy or waited at home to see if they improved, most did so because they considered the illness to be self-limited (41.5%) or because “the illness was not serious” (32.3%). Of those who stayed home and/or self-medicated, less than a quarter eventually sought care (23.9%). Even then, most of them (76.1%) waited “a few days” before seeking care. This is concerning because a major benchmark for the malaria program is to diagnose all active malaria cases within the first 48 hours of symptom onset. Additional efforts are needed to ensure that people link the need for malaria testing with onset of cardinal symptoms such as fever/chills as well as strengthen community-based testing interventions such as active surveillance by trained CHWs.

That most people either downplay potential malaria infection, do not link cardinal symptoms to possible infection, and/or delay care-seeking may be due to a few key reasons. First, a considerable portion of the population face difficult economic circumstances and likely prioritizes other life demands above the risk of malaria infection, which is still relatively low compared with other health issues. The impact of COVID-19-related restrictions on daily activities (such as government-mandated evening curfews) has likely forced people to make difficult decisions about personal health and the need to generate household income. Furthermore, people may view cardinal symptoms as simply not worth the trouble of alerting local CHWs or the financial expense of visiting crowded clinics or hospitals. Under these circumstances, malaria is likely not seen as a major problem in daily life. Another contributing factor to low levels of timely contact with the health system may be due to weak or strained relationships between community members and those whom they are encouraged to alert when sick with fever. The quality of the relationship between community members and community health workers is the focus of an ongoing ethnographic study. Going forward, the malaria program will have to strengthen its community engagement approach so that, on the one hand, it acknowledges the everyday reality of people who contend with multiple challenges aside from malaria; on the other hand, it must continue to emphasize the crucial role of community members in both controlling outbreaks and working toward elimination, particularly by alerting CHWs of illness, however mild symptoms may be.^19^

In this vein, community health concerns should be folded into malaria interventions. Rather than attempt to convince communities of how much a problem malaria is (at least for the field staff and professionals trying to eliminate the parasite), engagement activities should emphasize three public health messages: 1) malaria is a serious, potentially fatal disease; 2) treatment is available at local health facilities; and 3) individuals can take concrete steps to reduce mosquitos in their neighborhoods. For example, in neighboring Haiti, a community-directed larval source management project helped foster more trust within the community to allow for increased surveillance, identified key individuals as important focal persons for scaling up malaria control activities, and created more spaces for community dialogue.^20^ Community-based interventions for malaria, such as MDA, can include screening for diabetes and hypertension,^21^ which would demonstrate to community members how the malaria program recognizes their concerns. Further discussion of MDA strategies in this setting are published elsewhere.^15^

These findings also reveal the need to strengthen links between the malaria program and clinics and hospitals in transmission foci. Most participants (63%) expressed a clear preference for higher level care centers should they fall ill with malaria symptoms. At the same time, only 7.6% named medical doctors as primary sources of information related to malaria, although overall, few people ultimately sought care at hospitals and clinics. Nonetheless, this still reveals a potential opportunity for the medical establishment to raise awareness about malaria in clinical and community settings. Most people seem to learn about malaria through their friends, family, or television. In contrast to a rapid ethnographic study done in the same area in 2019,^15^ CHWs were not as frequently cited as sources of information about malaria (mentioned by only 14.3%). This presents an unusual paradox: ethnographic work has found that many patients are misdiagnosed in clinical settings and later diagnosed in the community, leading them to look more favorably upon CHWs and others doing active surveillance.^8^^,^^17^ At the same time, residents clearly prefer to seek medical care at tertiary-level centers when sick with fever and expect the involvement of medical professionals in potential MDA.^15^ Ongoing ethnographic work continues to explore these issues in more depth, including how the COVID-19 pandemic has affected the malaria program and people’s willingness to engage in antimalaria activities.

There are limitations to this study. Some practicalities may have introduced bias into the sample. First, the sampling frame was modified by eliminating four barrios (two from each focus) because of logistic and safety concerns. These four barrios had low malaria incidence (range: 0.18–0.32), so their removal from the sampling frame may have contributed to underrepresentation of lower incidence barrios. Second, surveying could not be done when most residents were presumed to be home (late evenings) because of safety reasons and the country’s mandatory COVID-19 curfew. Third, designating reference points within each barrio were based on logistics and team familiarity with the barrios (although one of four reference points was randomly eliminated). Fourth, since malaria incidence was higher in Los Tres Brazos than La Ciénaga, more barrios were selected within that transmission focus, which led to disproportionate representation between the two foci. One barrio within the La Ciénaga focus was oversampled by 10 participants.

Nearly half the sample (42.8%) was unable to recall last fever or care practices for last fever; caution is therefore needed when interpreting results from that portion of the questionnaire. A helpful approach to address this shortcoming would be to initiate in-depth case studies in hospitals, clinics, and neighborhoods to gain a better understanding of how fever is understood in relation to malaria and how decisions are made to seek care. Desirability bias is likely reflected in discrepancies between proportions of responses to some questions. For example, few participants named stagnant water as a potential cause of malaria, yet later, most said that there was a relationship between stagnant water and malaria.

There are always conceptual issues with KAP questionnaires. By their nature, KAP questionnaires essentially test people about their knowledge of specific biomedical facts. The biomedical language, phrasing, and answer choices of these questionnaires do not always correspond to people’s local understandings, subjective experiences, and strategies for dealing with malaria.^22^ For these reasons, the study team has complemented this study with a longitudinal, ethnographic project based on repeated in-depth interviews with key informants in both foci. Analysis and sharing of the results from that branch of the research project is forthcoming.

## CONCLUSION

Malaria outbreaks in Santo Domingo pose a new and complicated challenge for malaria elimination in the Dominican Republic and on the island as a whole. The findings shared here emphasize the need to strengthen community-based interventions in both foci—especially in high-incidence barrios of Los Tres Brazos, despite long-standing activities carried out by the malaria program in this area. Ensuring earlier diagnosis and treatment of the local population requires supporting active surveillance teams and fostering their acceptance in communities. Community engagement will therefore be essential to earn the trust of residents, increase their interest in malaria control and elimination, and support them going forward. This will require integrating malaria elimination with other local health and development priorities.

## Supplemental files


Supplemental materials
