# Supplementary material for: Results from a Knowledge, Attitudes, and Practices Survey in Two Malaria Transmission Foci of Santo Domingo, Dominican Republic
Source: Am J Trop Med Hyg. 2023 Feb 27;108(4):755–67. doi: 10.4269/ajtmh.22-0346 (PMC10077019; doi:10.4269/ajtmh.22-0346)
Supplement: Supplementary file 1 [file tpmd220346.SD1.pdf]

Investigación de conocimiento, actitudes, y prácticas sobre la malaria en Santo Domingo, Republica Dominicana

Investigation of knowledge, attitudes, and practices of malaria in Santo Domingo, Dominican Republic

Diciembre, 2020

Número del encuestador(a) **(surn)**
ID number of surveyor
1
2
3
4
5
6
7
8

Fecha **(date)**
Date

Hora de empezar **(stime)**
Time

Número de barrio **(clusnum)**
[rango, 1-23]

Nombre de barrio **(clusnam)**
[nombre del barrio]

Número de la encuesta **(suvnum)**
[integer]

Introducción
Introduction

Estoy aquí para hacer una encuesta en esta comunidad. Formo parte de un equipo más grande del Ministerio de Salud. Estamos visitando comunidades en Santo Domingo que han sido afectadas por la malaria. Me gustaría hacerle algunas preguntas rápidas y explicar el propósito de la encuesta. Luego, si lo desea, puede optar por participar. ¿Puedo hacerte algunas preguntas?

I am here to do a survey in this community. I am part of a larger team from the Ministry of Health. We are visiting communities in Santo Domingo that have been affected by malaria. I

would like to ask you a few quick questions and explain the purpose of the survey. Then, if you wish, you can choose to participate. Can I ask you a few questions?

Elegibilidad
Eligibility Screening

1. ¿Habla español? (**spa**)
   Do you speak Spanish?
   Si **1** [*Sigue a la siguiente pregunta*]
   Yes **1** [*Go to next question*]
   No **0** **[***Deténgase. Agradezca a la persona por su tiempo. Pasar a la siguiente casa***]**
   No **0** [*Stop. Thank person for their time. Proceed to next house*]
2. ¿Tiene al menos 18 años?
   Are you at least 18 years old? **(elig)**
   Si **1** [*Sigue a la siguiente pregunta*]
   Yes **1** [*Go to next question*]
   No **0** **[***Deténgase. Agradezca a la persona por su tiempo. Pasar a la siguiente casa***]**
   No **0** [*Stop. Thank person for their time. Proceed to next house*]
3. ¿Vive usted en esta casa?
   Do you live in this house? (**res**)
   Si **1** [*Sigue a la siguiente pregunta*]
   Yes **1** [*Go to next question*]
   No **0** **[***Deténgase. Agradezca a la persona por su tiempo. Pasar a la siguiente casa***]**
   No **0** [*Stop. Thank person for their time. Proceed to next house*]
4. ¿Duerme usted en esta casa todas las noches de la semana?
   Do you sleep at this house every night of the week? (**sleep**)
   Si **1** [*Sigue a la siguiente pregunta*]
   Yes **1** [*Go to next question*]
   No **0** **[***Deténgase. Agradezca a la persona por su tiempo. Pasar a la siguiente casa***]**
   No **0** [*Stop. Thank person for their time. Proceed to next house*]

Consentimiento
Consent script

Gracias por su interés en nuestro estudio sobre la malaria y la vida en su comunidad. Nos gustaría contarle todo lo que necesita pensar antes de decidir si participa o no al estudio. Es totalmente tu decisión. Si decide participar, puede cambiar de opinión más adelante y retirarse del estudio de investigación.

El objetivo del estudio es ayudar al Ministerio de Salud a desarrollar programas de salud. Queremos aprender de usted y otros miembros de su comunidad sobre sus experiencias y

opiniones relacionadas con la malaria. Para ello, este equipo de investigación está preguntando a personas de diferentes comunidades sobre su conocimiento sobre la malaria, qué hacen para prevenirla y cómo pueden recibir atención. También estamos interesados en otros temas, como la confianza en la comunidad, su sustento y seguridad, y seguridad financiera.

El objetivo del estudio es ayudar al Ministerio de Salud a desarrollar programas de salud. Su participación es voluntaria. Puede detenerse en cualquier momento. No se le pagará. No hay castigo si decide no participar. Sus respuestas se mantendrán confidenciales y no habrá forma de utilizar la información que comparte con nosotros para encontrarlo y hacerle daño posteriormente.

La encuesta tomará aproximadamente de una hora en completarse.

Puede tomar tanto tiempo como mínimo para responder las preguntas. También puede hacerme preguntas durante la encuesta. Usaré una tableta de mano para registrar sus respuestas. No grabaré su nombre.

Si se inscribe, se le pedirá que conteste preguntas sobre su vida, como su trabajo, lo que hace cuando esté enfermo, lo que puede saber sobre la malaria, la confianza en la comunidad y si se siente seguro.

Este estudio no está diseñado para beneficiarlo directamente. Este estudio está diseñado para aprender más sobre cómo personas como usted y como los miembros de esta comunidad comprenden y tratan el paludismo. También nos ayuda a comprender su vida diaria. Los resultados del estudio pueden usarse para ayudar a otros en el futuro.

Si tiene preguntas sobre este estudio, su participación en él, o si tiene preguntas o inquietudes sobre la investigación, puede comunicarse con los jefes de la investigación.

Thank you for your interest in our study about malaria and life in your community. We would like to tell you everything you need to think about before you decide whether or not to join the study. It is entirely your choice. If you decide to take part, you can change your mind later on and withdraw from the research study.

The goal of the study is to help the Ministry of Health develop health programs. We want to learn from you and other members of your community about your experiences and opinions related to malaria. To do this, this research team is asking people in different communities about their knowledge of malaria, what they do to prevent it, and how they are able to get care. We are also interested in other issues, like trust in the community, your livelihood and security.

Your participation is voluntary. You can stop at any time. You will not be paid. There is no punishment if you choose not to participate. Your responses will be kept confidential and there will be no way to use the information you share with us to later find and do harm to you.

The survey will take approximately 1 hour to complete.

You can take as long or as short to answer the questions. You may also ask me questions during the survey too. I will use a handheld tablet to record your answers. I will not record your name or your voice.

This study is not designed to benefit you directly. We are conducting over 400 of these interviews with people across the city. This study is designed to learn more about how people such as yourself and those in this community understand and deal with malaria. It also helps us understand daily life in your community. The study results may be used to help others in the future.

If you have questions about this study, your part in it, or if you have questions, or concerns about the research you may contact the head of the investigation.

5. ¿Está de acuerdo para participar en esta encuesta? (**cons**)
   Do you give consent to participate in this survey?
   - Si **1** [*Sigue a la siguiente pregunta*]
   - Yes **1** [*Go to next question*]
   - No **0** **[***Deténgase. Agradezca a la persona por su tiempo. Pasar a la siguiente casa***]**
   - No **0** [*Stop. Thank person for their time. Proceed to next house*]

Información demográfica
Demographic Information

Primero, le haré algunas preguntas sobre usted y las personas que viven en su casa.
First, I will ask you some questions about yourself and the people living in your house.

6. *¿La persona que contesta es masculino o femenino?* **(sex)**
   Is the participant male or female?
   Masculino **1**
   Male **1**
   Femenino **2**
   Female **2**

7. ¿Cuánto tiempo lleva usted viviendo aquí, en este barrio? *Escriba el tiempo en años; si es menos de 1 año, escriba 88; si no lo sabe, escriba 99.* (**rest**)
   How long have you lived here, in this neighborhood? *Write time in years; if less than 1 year, write 88; if don't know, write 99.*

   ______

8. Piensa en una semana típica. ¿Cuántas personas duermen en esta casa todas las noches? (**quanh**)
   Think of a typical week. How many people sleep in this house every night?

_____

9. ¿Cuantos años tiene usted? *Escribe la edad en años*. (**age**)
   How old are you? *Write age in years*.

_____

10. ¿Cuál es su estado civil? (**civ**)
    What is your civil status?
    Casado/a **1**
    Married **1**
    Soltero/a **2**
    Single **2**
    Union libre **3**
    Civil union **3**
    Divorciado/a **4**
    Divorced **4**
    Viudo/-a **5**
    Widowed **5**
    No sabe **99**
    Don't know **99**

11. ¿Á que nivel llegó de educación? **(edu)**
    What is the highest level of education you completed?
    Primero incompleto **1**
    Some primary **1**
    Primario completo **2**
    Primary (complete) **2**
    Secundario incompleto **3**
    Some secondary **3**
    Secundario completo **4**
    Secondary (complete) **4**
    Más que secundario **5**
    Above secondary **5**
    Nada **6**
    None **6**
    No sabe **99**
    Don't know **99**

12. ¿En que trabaja, o á que dedica? *No lea las respuestas*. **(occ)**
    What is your main way to earn money? *Do not read answers.*
    comerciante **1**
    store owner **1**
    ama de casa **2**
    homemaker **2**
    construcción **3**

construction **3**
chiripero **4**
informal work **4**
estudiante **5**
student **5**
lotería / banquera **6**
lottery ticket sales **6**
moto-concho/taxista **7**
motorcycle taxi/driver **7**
guardiana **8**
security guard **8**
Estilista **9**
Stylist / beauty store **9**
retirado **10** **[***Sigue a la 15]*
retired **10** *[Skip to 15]*
no trabaja **77** **[***Sigue a la 15]*
unemployed **77** *[Skip to 15]*
otros **88**
Other **88**
No sabe **99** *[Skip to 15]*
Don't know **99** *[Skip to 15]*

13. ¿A que hora empieza usted el trabajo normalmente? *Elige una sola respuesta.* **(works)**
What time of the day do you usually start working? *Select one.*
3:00-5:59 de la mañana/in the morning **1**
6:00-8:59 de la mañana/in the morning **2**
9:00-11:59 de la mañana/in the morning **3**
12:00-2:59 en la tarde/in the afternoon **4**
3:00-5:59 en la tarde/in the afternoon **5**
6:00 -8:59 en la noche/at night **6**
9:00-11:59 en la noche/at night **7**
Depende **8**
No consistent pattern **8**
No sabe **99**
Don't know **99**

14. ¿A que hora termina usted el trabajo normalmente? *Elige una sola respuesta.* **(workf)**
What time of the day do you usually stop working? *Select one.*
3:00-5:59 de la mañana/in the morning **1**
6:00-8:59 de la mañana/in the morning **2**
9:00-11:59 de la mañana/in the morning **3**
12:00-2:59 en la tarde/in the afternoon **4**
3:00-5:59 en la tarde/in the afternoon **5**
6:00 -8:59 en la noche/at night **6**
9:00-11:59 en la noche/at night **7**
Depende **8**

No consistent pattern **8**
No sabe **99**
Don't know **99**

Prácticas sobre fiebre y buscar atención
Fever and care-seeking practices

Ahora me gustaría hacerle algunas preguntas sobre estar enfermo y recibir atención.
Now I would like to ask you some questions about being sick and getting care.

15. ¿Cuáles son los tres problemas mas grandes de salud en su barrio? *Puede seleccionar solo uno.*
    What are the three most serious health problems in your neighborhood? *May select only one.*

15a. Problema salud 1 (heac1)
Health Problem 1
Malaria **1**
Malaria **1**
Dengue **2**
Dengue **2**
Presión **3**
Hypertension **3**
Diabetes/azúcar **4**
Diabetes **4**
Cancer **5**
Cancer **5**
Accidentes / heridas / trauma **6**
Accidents/injuries/trauma **6**
Fiebre **7**
Fever **7**
Gripe **8**
Common cold/flu **8**
Covid **9**
Covid **9**
Diarrhea/GI infection **10**
Diarrea/infecciones gastrointestinales **10**
Skin rashes / skin infection **11**
Erupciones de piel / infecciones de piel **11**
VIH/ SIDA **12**
HIV / AIDS **12**
ETS (enfermedad de transmisión sexual) **13**
STDs **13**
Otro **88**
Other **88**
No sabe **99**
Don't know **99**

15b. Problema salud 2 (heac2)
Health Problem 2
Malaria **1**
Malaria **1**
Dengue **2**
Dengue **2**
Presión **3**
Hypertension **3**
Diabetes/azúcar **4**
Diabetes **4**
Cancer **5**
Cancer **5**
Accidentes / heridas / trauma **6**
Accidents/injuries/trauma **6**
Fiebre **7**
Fever **7**
Gripe **8**
Common cold/flu **8**
Covid **9**
Covid **9**
Diarrhea/GI infection **10**
Diarrea/infecciones gastrointestinales **10**
Skin rashes / skin infection **11**
Erupciones de piel / infecciones de piel **11**
VIH/ SIDA **12**
HIV / AIDS **12**
ETS (enfermedad de transmisión sexual) **13**
STDs **13**
Otro **88**
Other **88**
No sabe **99**
Don't know **99**

15c. Problema salud 3 (heac3)
Health Problem 3
Malaria **1**
Malaria **1**
Dengue **2**
Dengue **2**
Presión **3**
Hypertension **3**
Diabetes/azúcar **4**
Diabetes **4**
Cancer **5**
Cancer **5**

Accidentes / heridas / trauma **6**
Accidents/injuries/trauma **6**
Fiebre **7**
Fever **7**
Gripe **8**
Common cold/flu **8**
Covid **9**
Covid **9**
Diarrhea/GI infection **10**
Diarrea/infecciones gastrointestinales **10**
Skin rashes / skin infection **11**
Erupciones de piel / infecciones de piel **11**
VIH/ SIDA **12**
HIV / AIDS **12**
ETS (enfermedad de transmisión sexual) **13**
STDs **13**
Otro **88**
Other **88**
No sabe **99**
Don't know **99**

16. ¿Qué campañas de salud pública organizadas por el Ministerio de Salud se implementan normalmente en su barrio? *Puede escoger mas que uno.*
What public health campaigns are done in your neighborhood? *May select more than one.*
(healthpro)
Malaria **1**
Dengue **2**
Chikungunya **3**
Rabies **4**
Vaccinations **5**
Zika **6**
COVID **7**
Maternal care **8**
Nutrition **9**
Teenage pregnancy **10**
Diabetes **11**
Hypertension **12**
Other **88**
None **77**
Don't know **99**

Ahora, piense en la última vez que tuvo fiebre.
Now, think about the last time you had fever.

17. ¿Qué hizo primero? *Seleccione la primera respuesta.*

What did you do first? *Select first response.* (**fev**)
Esperé a ver si mejoro **1** **[***Sigue a la 17a***]**
Waited to see if I get better **1** [*Go to 17a*]
Tomé diclofenaco, acetaminophen o jarabe en casa **2** [*Go to 17a*]
Took diclofenac, acetaminophen or *jarabe* at home **2** [*Go to 17a*]
Fui de inmediato a la clínica o hospital **3** **[***Sigue a la 18]*
Went immediately to the clinic or hospital **3** *[Go to 18]*
Llamé la promotora o colcom **4** **[***Sigue a la 18]*
Call the *promorotra* or *colcom* **4** *[Go to 18]*
No sabe **99** *[Sigue a la 20]*
Don't know **99** *[Go to 20]*

17a. ¿Por qué esperó en casa con fiebre? *Seleccione la primera respuesta.*
Why did you wait at home with fever? *Select first response.* **(rewait)**
A ver si me recupero **1**
To see if I get better **1**
La enfermedad no era grave **2**
The illness was not serious **2**
No pude faltar al trabajo **3**
I could not miss work **3**
Era demasiado caro para ir al medico **4**
It was too expensive to see the doctor **4**
No me gusta el medico/hospital/ la clínica **5**
I do not like the doctors/hospital/clinic **5**
Otro **88**
Other **88**
No sabe **99**
Don't know **99**

17b. Finalmente, ¿buscó atención?
Eventually, did you seek care? **(fevcare)**
Si **1** [*Sigue a la 17b1*]
Yes **1** [*Go to 17b1*]
No **0**
No **0**
No sabe **99**
Don't know **99**

17b1. Antes de buscar atención, ¿cuánto tiempo esperó en casa? *Lee las respuestas.*
Before seeking care, how long did you wait at home? *Read the answers.*
(waittime)
Algunos días **1**
A few days **1**
Mas que una semana **2**
More than 1 week **2**
Otro **88**

Other **88**
No sabe **99**
Don't know **99**

17b2. Una vez que buscó atención para la fiebre, ¿a dónde fue? *Puede seleccionar solo uno.*
Once you sought care for fever, where did you go? *May select only one.***(care)**
Unidad de atención primaria (UNAP) **1**
Primary care clinic (UNAP) **1**
Casa de colcom **2**
House of *colcom* **2**
Casa de promotora **3**
House of *promotora* **3**
Hospital **4**
Hospital **4**
Otro **88**
Other **88**
No sabe **99**
Don't know **99**

17b3. ¿Era difícil para ti ir allí?
Was it hard for you to go there? **(carebar)**
Si **1** [*Sigue a la 17b3i.*]
Yes **1** [*Go to 17b3i.*]
No **0**
No **0**
No sabe **99**
Don't know **99**

17b3i. ¿Por qué le resultó difícil buscar atención? *Seleccione la primera respuesta.* **(rebar)**
Why was it hard for you to seek care? *Select the first response.*
No pude faltar al trabajo **1**
I could not miss work **1**
Debí cuidar de mi familia **2**
I had to take care of my family **2**
No pude pagar el costo de transporte **3**
I could not pay for transport **3**
No tengo seguro **4**
I do not have insurance **4**
Los médicos no me atienden / no saben nada **5**
The doctors do not help me / do not know anything **5**
La promotora no me ayuda / no tengo confianza en la promotora **6**
The *promotora* cannot help me / I do not trust the promotor **6**
El colcom no me ayuda / No tengo confianza en el colcom **7**
The *colcom* cannot help me / I do not trust the colcom **7**

Otro **88**
Other **88**
No sabe **99**
Don’t know **99**

18. ¿Se realizó una prueba de sangre? (**bloodfev**)
Did they do a blood test?
Si **1**
Yes **1**
No **0**
No **0**
No sabe **99**
Don’t know **99**

19. ¿Le han dicho que tenía malaria? (**fevmal**)
Did they tell you that you have malaria?
Si **1** *[Sigue a la 19a]*
Yes **1** *[Go to 19a]*
No **0**
No **0**
No sabe **99**
Don’t know **99**

19a. ¿Tomó medicina? (**fevmed**)
Did you take medicine?
Si **1** *[Sigue a la 19a1]*
Yes **1** *[Go to 19a1]*
No **0**
No **0**
No sabe **99**
Don’t know **99**

19a1. ¿Mejoró?
Did you get better? (**fevbet)**
Si **1**
Yes **1**
No **0**
No **0**
No sabe **99**
Don’t know **99**

20. ¿Alguien ha visitado alguna vez su casa para hacer una prueba de malaria?
Has anyone ever visited your house to do a malaria test? **(bloodtest)**
Si **1** *[Sigue a la pregunta 20a]*
Yes **1** *[Go to question 20a]*
No **0** *[Sigue a la pregunta 21]*

No **0** *[Go to question 21]*
No sabe **99** *[Sigue a la pregunta 21]*
Don't know **99** *[Go to question 21]*

20a. ¿Cuando? *Lee las respuestas.* **(whenblood)**
When? *Read the answers.*
Dentro de la misma semana **1**
Within the same week **1**
La semana pasada **2**
Last week **2**
Hace algunas semanas **3**
A few weeks ago **3**
Hace mas que un mes **4**
More than a month ago **4**
Hace mas que algunos meses **5**
More than a few months ago **5**
No sabe **99**
Don't know **99**

20b. ¿Aceptó la prueba de malaria?
Did you accept the malaria test? (**bloodac**)
Si 1
Yes **1**
No **0** *[Sigue a la 20b.i]*
No **0** *[Go to 20b.i]*
No sabe **99**
Don't know **99**

20b.i. ¿Porqué no? *No lee las respuestas*
Why not? **(bloodref)**
No me gusta / duele mucho **1**
I do not like it / it hurts **1**
No confío en ellos **2**
I do not trust them **2**
No es útil / no sirve a nada **3**
It is not helpful / doesn't do anything **3**
No quiero padecer de COVID / Riesgo de COVID **4**
I do not want to get sick from COVID / risk of COVID **4**
Other (write response) **88**
Don't know **99**

21. En cualquier momento del pasado ¿Le han dicho alguna vez que tiene malaria?
At any time in the past, have you ever been told that you have malaria? (**havemal**)
Si **1** [*Sigue a 21a*]
Yes **1** [*Go to 21a*].
No **0** *[Sigue a la 22]*

No **0** *[Go to 22]*
No sabe **99** *[Sigue a la 22]*
Don't know **99** *[Go to 22]*

21a. ¿Quien le lo dijo?
Who told you? (**malwho1**)
Promotora/colcom **1**
Promotora/colcom **1**
Visita en casa por salud publica **2**
House visit by public health **2**
Medico en la UNAP o hospital **3**
Clinic doctor **3**
Other **88**
Otro **88**
No sabe **99**
Don't know **99**

21b. ¿Le hicieron un análisis de sangre?
Did you get your blood tested? (**maldx1)**
Si **1**
Yes **1**
No **0**
No **0**
No sabe **99**
Don't know **99**

21c. ¿Tomó la medicina?
Did you take medicine? **(takemed)**
Si **1** *[Sigue a la 21c1]*
Yes **1** *[Go to 21c1]*
No **0**
No **0**
No sabe **99**
Don't know **99**

21c1. ¿Se mejoró?
Did you get better? **(medbet)**
Si **1**
Yes **1**
No **0**
No **0**
No sabe **99**
Don't know **99**

Conocimiento y prevención de malaria

Malaria knowledge and prevention

Ahora me gustaría hacerle preguntas sobre la malaria y las formas en que puede tratar de prevenirla.
Now I would like to ask you questions about malaria and ways you may try to prevent it.

22. ¿Ha oído hablar alguna vez del problema de malaria en la capital? **(malk)**
    Have you ever heard of malaria in the capital?
    Si **1**
    Yes **1**
    No **0**
    No **0**
    No sabe **99**
    Don't know **99**

23. ¿Cómo se enferma la gente de malaria? *Puede seleccionar más de uno.*
    How do people get sick from malaria? *May select more than one.* **(malsick1-4)**
    Picadura del mosquito **1**
    Mosquito bites **1**
    Basura **2**
    Trash **2**
    Cañada / agua estancada **3**
    Canal / stagnant water **3**
    Comer caña dañada **4**
    Eating bad sugarcane **4**
    Comer maíz **5**
    Eating maize **5**
    Respirar polvo **6**
    Inhaling pollen/dust **6**
    Hambre **7**
    Hunger **7**
    Comer alimento contaminado **8**
    Eating dirty food **8**
    Tomar agua contaminada **9**
    Drinking dirty water **9**
    Mojarse en la lluvia **10**
    Getting soaked with rain **10**
    Tiempo de frío o cambio de clima **11**
    Cold or changing weather **11**
    Brujería / espíritu malo **12**
    Witchcraft **12**
    Tomar leche **13**
    Drinking milk **13**
    Comer chivo **14**
    Eating goats meat **14**
    Dormir en el suelo mojado **15**

Sleeping on wet ground **15**
Mucho trabajo **16**
Increased workload **16**
Falta higiene **17**
Poor personal hygiene **17**
Tomar sol **18**
Too much sun **18**
Bañarse en el río **19**
Swim in river **19**
Otro **88**
Other **88**
No sabe **99**
Don't know **99**

24. ¿Cuáles son los síntomas de la malaria? *Puede elegir más de uno.*
What are the symptoms of malaria? *May choose more than one.*
(**mals**)
La fiebre **1**
Fever **1**
Escalofrío **2**
Chills **2**
Dolor de cabeza **3**
Headache **3**
Nauseas/vómitos **4**
Nausea/Vomiting **4**
Falta de apetito **5**
Loss of appetite **5**
Dolor en el cuerpo, dolor articulaciones **6**
Body ache or joint pain **6**
Perdido del conocimiento **7**
Loss of consciousness **7**
Ictericia **8**
Jaundice **8**
Otros **88**
Other **88**
No sabe **99**
Don't know **99**

25. Si tiene esos síntomas, ¿que es lo primero que haría?
If you have these symptoms, what is the first thing you do? **(gettest)**
Llama a la promotora **1**
Call the promotora **1**
Llama al colcom **2**
Call the colcom **2**
Va a la UNAP **3**

Go to UNAP **3**
Va al hospital **4**
Go to hospital **4**
Espera para ver si mejoro **5**
Wait to see if I get better **5**
Otro **88**
Other **88**
No sabe **99**
Don't know **99**

26. ¿Cómo se puede prevenir la malaria? *Puede elegir más de uno.*
How can someone prevent malaria? *May choose more than one.* (**malprev1-8**)
Dormir debajo del mosquitero **1**
Sleep under mosquito net **1**
Usa repelente contra la picadura del mosquito **2**
Use mosquito repellant **2**
Evitar la picadura del mosquito **3**
Avoid mosquito bites **3**
Tomar medicamentos de prevención contra la malaria (medicamento de prevención) **4**
Take preventative medicine **4**
Aplicar insecticida **5**
Spray house with insecticide **5**
Cortar las yerbas alrededor de la casa **6**
Cut grass around the house **6**
No dejar agua estancada alrededor de la casa **7**
Fill in puddles (stagnant water) **7**
Quemar hojas de los árboles en la casa **8**
Burn leaves **8**
No tomar agua sucia/contaminada **9**
Don't drink dirty water **9**
No usar comida contaminada **10**
Don't eat bad food **10**
Poner mallas a las ventanas **11**
Use window coverings/screens **11**
Comer ajo **12**
Eat garlic **12**
Nada puede prevenir la malaria **13**
Nothing can protect against malaria **13**
Tomar alcohol **14**
Drinking alcohol **14**
Comer pimienta **15**
Eating hot pepper **15**
No comer caña/maíz **16**
Not eating maize/sugarcane **16**
Orar a Dios **17**
Pray / God **17**

Lavarse las manos **18**
Wash hands **18**
Echar cloro **19**
Put chlorine in water **19**
Cubrir los tanques / tapas **20**
Cover tanks/containers closed **20**
Otros **88**
Other **88**
No sabe **99**
Don't know **99**

27. ¿A través de quien ha escuchado hablar de la malaria? *Puede elegir más de uno.*
How did you learn about malaria? *May choose more than one.* (**mallearn**)
Amigos **1**
Friends **1**
Familia / familiares **2**
Family **2**
Escuela **3**
School **3**
Visita domiciliara por la promotora, salud publica, o *colcom* **4**
House visit by public health/*promotora/colcom* **4**
Charla **5**
Educative talk in community/*charla* **5**
Clínica, doctor **6**
Clinic/doctor **6**
Radio **7**
Radio **7**
Television **8**
Television **8**
Redes sociales como Facebook, Instagram, WhatsApp **9**
Social networks like Facebook, Instagram, WhatsApp, etc **9**
Otro **88**
Other **88**
No sabe **99**
Don't know **99**

28. ¿En quién confía más para aprender sobre la malaria? *Puede elegir más de uno.*
Who do you trust the most to learn more about malaria? *May select more than one.*
(maltrust)
Amigos **1**
Friends **1**
Familia / familiares **2**
Family **2**
Escuela **3**
School **3**
Líder comunitario **4**

Community leader **4**
Presidente de la junta de vecinos **5**
President of *junta de vecinos* **5**
Visita domiciliara por la promotora, salud publica, o *colcom* **6**
House visit by public health/*promotora/colcom* **6**
Clínica, doctor **7**
Clinic / doctor **7**
Gobierno / estado **8**
Government **8**
Redes sociales como Facebook, Instagram, WhatsApp **9**
Social networks like Facebook, Instragram, WhatsApp, etc **9**
Otro **88**
Other **88**
No sabe **99**
Don't know **99**

29. Sabe como se detecta la malaria?
Do you know malaria is detected? **(maltest)**
Si **1** **[***Sigue a la 29a***]**
Yes **1** *[Go to 29a]*
No **0**
No **0**
No sabe **99**
Don't know **99**

29a. ¿Como? (**maltest1)**
Prueba / análisis de sangre / prueba rápida **1**
Test / blood analysis / rapid test **1**
Otro **88**
Other **88**
No sabe **99**
Don't know **99**

30. ¿Hay medicamentos para curar la malaria?
Are there medicines that can cure malaria? **(malcure)**
Si **1**
Yes **1**
No **0**
No **0**
No sabe **99**
Don't know **99**

31. ¿Tiene algo que ver el agua estancada con la malaria? *No lee las respuestas.*
Is there a relationship between stagnant water and malaria? *Don't read the answers.*
(watmal)
Si **1**

Yes **1**
No **0**
No **0**
No sabe **99**
Don't know **99**

Prácticas sobre la malaria
Malaria practices
Ahora, me gustaría hacerle preguntas sobre lo que usted o su familia pueden hacer para prevenir la malaria.
Now, I would like to ask you questions about what you or your family may do to prevent malaria.

32. ¿Usted o su familia hacen algo para solucionar el agua estancada?
Do you or your family do anything to address stagnant water? (**watdo**)
Si **1** *[Sigue a la pregunta 32a.]*
Yes **1** *[Go to question 32a.]*
No **0**
No **0**
No sabe **99**
Don't know **99**

32a. Que hace usted? (**watdo1**) *Puede escoger mas que uno.*
What do you do? *May select more than one.*
Rellenar hoyos **1**
Fill in holes **1**
Quitar gomas vacías **2**
Remove empty tires **2**
Cubrir recipientes de agua **3**
Cover containers **3**
Echar cloro **4**
Put chlorine **4**
Otro **88**
Other **88**
No sabe **99**
Don't know **99**

33. ¿Pueden los mosquiteros ayudar a detener la malaria?
Can mosquito nets help stop malaria? **(malnet)**
Si **1**
Yes **1**
No **0**
No **0**
No sabe **99**
Don't know **99**

34. ¿Tienen mosquiteros en su casa? **(net)**
Do you have mosquitos nets in your home?
Si **1** *[Sigue a la 34a]*
Yes **1** *[Go to 34a]*
No **0** *[Sigue a la 36]*
No **0** *[Go to 36]*
No sabe **99**
Don't know **99**

34a. ¿Cuantos mosquiteros tienen en la casa? (**netnum**)
How many mosquito nets do you have in the home?

_______ [integer]

34b. ¿Cuantas personas en la casa durmieron abajo esos mosquiteros anoche? (**netuseh1**)
How many people in your house slept under these nets last night?

_______ [integer] **[***IF LESS THAN Q8 "quanh" GO TO 34B.i***]:**

34b.i. ¿Por qué no todos dormían abajo un mosquitero anoche? **(renoneth)**
Why didn't everyone sleep under the net last night?
Demasiado calor **1**
It is too hot **1**
Mal olor **2**
It smells bad **2**
Está sucio **3**
It is dirty **3**
Está roto **4**
It is ripped / broken **4**
No les gustan **5**
They don't like it **5**
No hay suficiente espacio **6**
Not sufficient space **6**
Otras personas no estaban en casa anoche **7**
Other people weren't home last night **7**
Otro **88**
Other **88**
No sabe **99**
Don't know **99**

35. ¿Tiene mosquitero para usted? **(netself)**
Do you have a mosquito net for yourself?
Si **1** *[Sigue a la 35a]*
Yes **1** *[Go to 35a]*

No **0** *[Sigue a la 36]*
No **0** *[Go to 36]*
No sabe **99**
Don't know **99**

35a. ¿Dónde conseguiste el mosquitero?
Where did you get the net? **(netsource)**
Campaña / ONG **1**
Public health campaign / NGO **1**
Mercado / colmado / tienda **2**
Bought at market or store **2**
Familia / amigos / familiares **3**
Family/friend / relatives **3**
Hospital **4**
At hospital **4**
Otro **88**
Other **88**
No sabe **99**
Don't know **99**

35b. ¿Dormiste debajo de él anoche?
Did you sleep under it last night? **(netuse)**
Si **1**
Yes **1**
No **0** **[***Sigue a la 35b.i*
No **0** **[***Go to 35b.i]*
No sabe **99**
Don't know **99**

35b.i. ¿Por qué no?
Why not? **(renonnet)**
Demasiado calor **1**
It is too hot **1**
Mal olor **2**
It smells bad **2**
Está sucio **3**
It is dirty **3**
Está roto **4**
It is ripped / broken **4**
No me gusta **5**
I don't like it **5**
Otro **88**
Other **88**
No sabe **99**
Don't know **99**

36. ¿Ha visto alguna vez un equipo de salud pública fumigando dentro de las casas de su barrio?
Have you ever seen a team from public health spray the inside of houses in your neighborhood? **(irshome)**
Si **1**
Yes **1** *[Go to 36a]*
No **0**
No **0**
No sabe **99**
Don't know **99**

36a. ¿Permitió la fumigación en su casa? **(letspray)**
Did you allow the team to spray your house?
Si **1** **[***Sigue a la 36b]*
Yes **1** *[Go to 36b]*
No **0** *[Sigue a la 36a.1]*
No **0**
No sabe **99**
Don't know **99**

36a.1 Why did you not permit them to spray your home? (**nosprayh1-3**)
The spray smells bad **1**
The spray is not healthy **2**
The spray does not really work **3**
I do not trust it **4**
Proteger los niños en la casa **5**
To protect the children in the home **5**
I am afraid of COVID **6**
Other **88**
Don't know **99**

36b. ¿Cuando? *Lee las respuestas.* **(whenspray)**
When? *Read the answers.*
Dentro de la misma semana de la encuesta **1**
Within the same week of the survey **1**
La semana pasada **2**
Last week **2**
Hace algunas semanas **3**
A few weeks ago **3**
Hace mas que un mes **4**
More than a month ago **4**
Hace mas que algunos meses **5**
More than a few months ago **5**
No sabe **99**
Don't know **99**

37. Ahora, y considerando lo que ha sucedido con el problema de COVID, ¿está más o menos dispuesto a permitir que se haga una fumigación dentro de su casa? *Lee las respuestas.*
Right now, and considering what has happened with the COVID problem, are you more or less motivated to allow fumigation in your home? *Read the answers.* **(covirs)**
Muy animado **5**
Very active (motivated) **5**
Animado **4**
Motivated **4**
Normal **3**
Neutral **3**
Desanimado **2**
Unmotivated **2**
Muy desanimado/a **1**
Very unmotivated **1**
No sabe **99**
Don't know **99**

Actitudes de malaria
Malaria attitudes

Ahora me gustaría hacerle algunas preguntas generales sobre cómo ve el problema de la malaria.
Now I would like to ask you some general questions about how you see the problem of malaria.

38. ¿Es la malaria un problema aquí el barrio?
Is malaria a problem here in the neighborhood? **(malprob)**
Si **1**
Yes **1**
No **0**
No **0**
No sabe **99**
Don't know **99**

39. ¿La mayoría de las fiebres son malaria? **(fevmal1)**
Are most fevers malaria?
Si **1**
Yes **1**
No **0**
No **0**
No sabe **99**
Don't know **99**

40. Ahora escucha bien. **"Una MDA consiste en darle medicamentos para la malaria a toda la gente que vive en una zona con riesgo de contraer la enfermedad,**

independientemente que tenga o no síntomas, para lograr la eliminación de malaria.” Después de escuchar eso, pero considerando lo que ha sucedido con el problema de COVID, ¿está más o menos dispuesto a participar en una campaña de MDA contra la malaria planificada por el gobierno? *Lee las respuestas.*

Now listen well. "An MDA consists of giving malaria drugs to all people living in an area at risk of contracting the disease, regardless of whether they have symptoms or not, to achieve the elimination of malaria." After hearing that, but considering what has happened due to the COVID problem, are you more or less willing to participate in a MDA of anti-malaria drugs planned by the government? **(covmda)** *Read the answers.*

Muy animado **5**
Very active (motivated) **5**
Animado **4**
Motivated **4**
Normal **3**
Neutral **3**
Desanimado **2**
Unmotivated **2**
Muy desanimado/a **1**
Very unmotivated **1**
No sabe **99**
Don’t know **99**

41. Ahora, y considerando lo que ha sucedido con el problema del COVID, ¿hasta que punto se siente motivado a aceptar una vacuna contra el COVID si el gobierno dice que es segura? *Lee las respuestas.*
Right now, and considering what has happened with the COVID problem, how motivated are you to accept a vaccine for COVID if the government says it is safe? *Read the answers.* **(covvac)**

Muy animado **5**
Very active (motivated) **5**
Animado **4**
Motivated **4**
Normal **3**
Neutral **3**
Desanimado **2**
Unmotivated **2**
Muy desanimado/a **1**
Very unmotivated **1**
No sabe **99**
Don’t know **99**

Participación comunitaria y confianza
Community and trust

Ahora, me gustaría hacerle preguntas sobre el barrio y la confianza.
Now, I would like to ask you questions about your community and trust.

42. Por primero, ¿Se siente comoda/-o en su barrio? *Lee las respuestas.* **(safe)**
    First, do you feel safe in your neighborhood? *Read the answer choices.*
    Siempre **5**
    Always **5**
    La mayoría de veces **4**
    Most times **4**
    Algunas veces **3**
    Sometimes **3**
    Casi nunca **2**
    Almost never **2**
    Nunca **1**
    Never **1**
    No sabe **99**
    Don't know **99**

43. En su opinión, ¿cree que su barrio está limpio? *Lee las respuestas.*
    In your opinion, do you think your neighborhood is clean? *Read the answer choices.* **(clean)**
    Siempre **5**
    Always **5**
    La mayoría de veces **4**
    Most times **4**
    Algunas veces **3**
    Sometimes **3**
    Casi nunca **2**
    Almost never **2**
    Nunca **1**
    Never **1**
    No sabe **99**
    Don't know **99**

44. En su opinión, ¿cree que la basura se recoge a tiempo en su barrio? *Lee las respuestas.*
    In your opinion, how reliable is the trash collection in your neighborhood? *Read the answer choices.* **(trash)**
    Siempre **5**
    Always **5**
    La mayoría de veces **4**
    Most times **4**
    Algunas veces **3**
    Sometimes **3**
    Casi nunca **2**
    Almost never **2**
    Nunca **1**

Never **1**
No sabe **99**
Don’t know **99**

45. En su opinión, ¿cree que las personas se preocupan por los demás en su barrio? *Lee las respuestas.*
    In your opinion, do you think that people care about each other in your neighborhood? *Read the answer choices.* **(carecom)**
    Siempre **5**
    Always **5**
    La mayoría de veces **4**
    Most times **4**
    Algunas veces **3**
    Sometimes **3**
    Casi nunca **2**
    Almost never **2**
    Nunca **1**
    Never **1**
    No sabe **99**
    Don’t know **99**

Ahora, me gustaría hablar con usted sobre la confianza.
Now, I would like to ask you questions about trust.

46. ¿Cuanto confía en la gente de su barrio? *Lée las opciones de respuesta disponibles* **(trusc)**
    How much do you trust your neighbors? *Read the available answer choices.*
    Muy confiable **3**
    Very trustworthy **3**
    Algo confiable **2**
    Somewhat trustworthy **2**
    Poco confiable **1**
    A little trustworthy **1**
    Nada confiable **0**
    Untrustworthy **0**
    No sabe **99**
    Don’t know **99**

47. Desde el inicio del problema del COVID, ¿ha aumentado, se ha mantenido igual o ha disminuido su confianza en la gente de su barrio? *Léeles las opciones de respuesta disponibles.*
    Since the beginning of the COVID problem, has your trust in your neighbors gone up, stayed the same, or gone down? **(covcom)**
    Ha aumentado **3**
    Has gone up **3**
    Se ha mantenido igual **2**

Stayed the same **2**
Ha disminuido **1**
Gone down **1**
No sabe **99**
Don't know **99**

48. ¿Cuanto confía en las promotoras de salud de su barrio? *Léeles las opciones de respuesta disponibles.*
How much do you trust the health promoters in your neighborhod? *Read the available answer choices.* **(trustp)**
Muy confiable **3**
Very trustworthy **3**
Algo confiable **2**
Somewhat trustworthy **2**
Poco confiable **1**
A little trustworthy **1**
Nada confiable **0**
Untrustworthy **0**
No hay promotora en el barrio **88**
No promotora in neighborhood **88**
No sabe **99**
Don't know **99**

49. Desde el inicio del problema del COVID, ¿ha aumentado, se ha mantenido igual o ha disminuido su confianza en las promotoras? *Léeles las opciones de respuesta disponibles.*
Since the beginning of the COVID problem, has your trust in the health promoters gone up, stayed the same, or gone down? **(covprom)**
Ha aumentado **3**
Has gone up **3**
Se ha mantenido igual **2**
Stayed the same **2**
Ha disminuido **1**
Gone down **1**
No hay promotora en el barrio **88**
No promotora in neighborhood **88**
No sabe **99**
Don't know **99**

50. **[SI APLICA A BARRIO]:** ¿Cuanto confía en el *colcom* de su comunidad? *Léeles las opciones de respuesta disponibles.*
[IF APPLICABLE TO BARRIO]: How much do you trust the *colcom* in your community? *Read the available answer choices.* **(trustcol)**
Muy confiable **3**
Very trustworthy **3**
Algo confiable **2**
Somewhat trustworthy **2**

Poco confiable **1**
A little trustworthy **1**
Nada confiable **0**
Untrustworthy **0**
No sabe **99**
Don't know **99**

51. **[SI APLICA A BARRIO]:** Desde el inicio del problema del COVID, ¿ha aumentado, se ha mantenido igual o ha disminuido su confianza en *el colcom*?
[IF APPLICABLE TO BARRIO]: Since the beginning of the COVID problem, has your trust in the *colcom* gone up, stayed the same, or gone down? **(covcol)**
Ha aumentado **3**
Has gone up **3**
Se ha mantenido igual **2**
Stayed the same **2**
Ha disminuido **1**
Gone down **1**
No sabe **99**
Don't know **99**

52. ¿Cuanto confía en el personal de la UNAP? *Léeles las opciones de respuesta disponibles.*
How much do you trust the personnel in the UNAP? *Read the available answer choices.*
(trustunap)
Muy confiable **3**
Very trustworthy **3**
Algo confiable **2**
Somewhat trustworthy **2**
Poco confiable **1**
A little trustworthy **1**
Nada confiable **0**
Untrustworthy **0**
No sabe **99**
Don't know **99**

53. Desde el inicio del problema del COVID, ¿ha aumentado, se ha mantenido igual o ha disminuido su confianza en el personal de la UNAP?
Since the beginning of the COVID problem, has your trust in the personnel in the UNAP gone up, stayed the same, or gone down? **(covunap)**
Ha aumentado **3**
Has gone up **3**
Se ha mantenido igual **2**
Stayed the same **2**
Ha disminuido **1**
Gone down **1**
No sabe **99**
Don't know **99**

54. ¿Cuanto confía en el personal del hospital público? *Léeles las opciones de respuesta disponibles.*
    How much do you trust the personnel in the public hospital? *Read the available answer choices.* **(trusthos)**
    Muy confiable **3**
    Very trustworthy **3**
    Algo confiable **2**
    Somewhat trustworthy **2**
    Poco confiable **1**
    A little trustworthy **1**
    Nada confiable **0**
    Untrustworthy **0**
    No sabe **99**
    Don't know **99**

55. Desde el inicio del problema del COVID, ¿ha aumentado, se ha mantenido igual o ha disminuido su confianza en el personal del hospital público?
    Since the beginning of the COVID problem, has your trust in the personnel in the public hospital gone up, stayed the same, or gone down? **(covhosppub)**
    Ha aumentado **3**
    Has gone up **3**
    Se ha mantenido igual **2**
    Stayed the same **2**
    Ha disminuido **1**
    Gone down **1**
    No sabe **99**
    Don't know **99**

56. ¿Cuanto confía en el personal en los centros privados? *Léeles las opciones de respuesta disponibles.*
    How much do you trust the personnel in private health centers? *Read the available answer choices.* **(trustpriv)**
    Muy confiable **3**
    Very trustworthy **3**
    Algo confiable **2**
    Somewhat trustworthy **2**
    Poco confiable **1**
    A little trustworthy **1**
    Nada confiable **0**
    Untrustworthy **0**
    No sabe **99**
    Don't know **99**

57. Desde el inicio del problema del COVID, ¿ha aumentado, se ha mantenido igual o ha disminuido su confianza en el personal en los centros privados? *Léeles las opciones de respuesta disponibles.*
Since the beginning of the COVID problem, has your trust in the personnel in the private health centers gone up, stayed the same, or gone down? **(covpriv)**
Ha aumentado **3**
Has gone up **3**
Se ha mantenido igual **2**
Stayed the same **2**
Ha disminuido **1**
Gone down **1**
No sabe **99**
Don't know **99**

58. ¿Cuanto confía el Ministerio de Salud? *Léeles las opciones de respuesta disponibles.*
How much do you trust the Ministry of Health? *Read the available answer choices.* **(trusm)**
Muy confiable **3**
Very trustworthy **3**
Algo confiable **2**
Somewhat trustworthy **2**
Poco confiable **1**
A little trustworthy **1**
Nada confiable **0**
Untrustworthy **0**
No sabe **99**
Don't know **99**

59. Desde el inicio del problema del COVID, ¿ha aumentado, se ha mantenido igual o ha disminuido su confianza en el Ministerio de Salud?
Since the beginning of the COVID problem, has your trust in the personnel in the Ministry of Health gone up, stayed the same, or gone down? **(covmin)**
Ha aumentado **3**
Has gone up **3**
Se ha mantenido igual **2**
Stayed the same **2**
Ha disminuido **1**
Gone down **1**
No sabe **99**
Don't know **99**

60. ¿Cuanto confía en la *junta de vecinos*? *Léeles las opciones de respuesta disponibles.*
How much do you trust the *junta de vecinos*? *Read the available answer choices.* **(trusj)**
Muy confiable **3**
Very trustworthy **3**
Algo confiable **2**

Somewhat trustworthy **2**
Poco confiable **1**
A little trustworthy **1**
Nada confiable **0**
Untrustworthy **0**
No sabe **99**
Don't know **99**

61. Desde el inicio del problema del COVID, ¿ha aumentado, se ha mantenido igual o ha disminuido su confianza en la *junta de vecinos*?
Since the beginning of the COVID problem, has your trust in the personnel in the *junta de vecinos* gone up, stayed the same, or gone down? **(covjdv)**
Ha aumentado **3**
Has gone up **3**
Se ha mantenido igual **2**
Stayed the same **2**
Ha disminuido **1**
Gone down **1**
No sabe **99**
Don't know **99**

62. ¿Cuanto confía en el gobierno nacional? *Léeles las opciones de respuesta disponibles.*
How much do you trust the national government? *Read the available answer choices.* **(trusg)**
Muy confiable **3**
Very trustworthy **3**
Algo confiable **2**
Somewhat trustworthy **2**
Poco confiable **1**
A little trustworthy **1**
Nada confiable **0**
Untrustworthy **0**
No sabe **99**
Don't know **99**

63. Desde el inicio del problema del COVID, ¿ha aumentado, se ha mantenido igual o ha disminuido su confianza en el gobierno nacional?
Since the beginning of the COVID problem, has your trust in the national government gone up, stayed the same, or gone down? **(covgov)**
Ha aumentado **3**
Has gone up **3**
Se ha mantenido igual **2**
Stayed the same **2**
Ha disminuido **1**
Gone down **1**
No sabe **99**

Don’t know **99**

64. Actualmente ¿Cómo califica la respuesta general del gobierno al COVID-19, según la escala siguiente: muy malo, malo, neutral, bueno, y muy bueno? *Léeles las respuestas.* How would you rate the response of the government to COVID-19, using the following scale: very poor, bad, neutral, good, and very good? *Read the answer choices.* **(covrat)**
Muy bueno **5**
Very good **5**
Bueno **4**
Good **4**
Neutral **3**
Neutral **3**
Malo **2**
Bad **2**
Muy malo **1**
Very poor **1**
No sabe **99**
Don’t know **99**

Ahora, me gustaría hacerle otras preguntas sobre su barrio.
Now, I would like to ask you other questions about your neighborhood.

65. Va usted a la iglesia? (**igl**)
Do you go to church?
Si **1** [*Sigue a 65a*]
Yes **1** **[***Go to 65a***]**
No **0**
No **0**
No sabe **99**
Don’t know **99**

65a. Que tipo de iglesia? (**relig)**
What kind of church? *Don’t read the answers.*
Católica **1**
Catholic **1**
Evangélica **2**
Evangelic **2**
Pentecostal **3**
Pentecostal **3**
Otro **88**
Other **88**
No sabe **99**
Don’t know **99**

65b. Antes del problema de COVID, con qué frecuencia asistía a la iglesia, u otras reuniones ligadas a la iglesia? *Léeles las respuestas.*

Before the problem of COVID, how often did you go to this church and/or attend church-related meetings? *Read the answers.* (**iglmeet**)
Más de una vez por semana **4**
More than once per week **4**
Una vez por semana **3**
Once a week **3**
Una o dos veces al mes **2**
Once or twice a month **2**
Una o dos veces al año **1**
Once or twice a year **1**
Nunca **0**
Never **0**
No sabe **99**
Don't know **99**

65c. Ahora, dado la situación de COVID, ¿Como ha cambiado su participación en la iglesia? *Lee las respuestas.* **(covigl)**
Now, given the COVID situation, how has your participation in church changed? *Read the answers.*
Igual **2**
The same **2**
Menor que antes **1**
Less than before **1**
Otro **88**
Other **88**
No sabe **99**
Don't know **99**

66. Aparte de las iglesias ¿ Hay otros grupos u organizaciones del barrio que conozca y que son activos en su barrio? (**group**)
Aside from churches, are there other neighborhood groups or organizations that you know about that are active in your neighborhood?
Si **1** [*Sigue a 66a*]
Yes **1 [***Go to 66a***]**
No **0**
No **0**
No sabe **99**
Don't know **99**

66a. Cuales? (*Puede escoger mas que uno*) (**grouptype1-4**)
Which ones? (*May choose more than one*)
Juntas de vecinos **1**
Neighborhood association **1**
Escuela **2**
School **2**
Partido político **3**

Political party **3**
Clubes deportivos **4**
Sport clubs **4**
Clubes culturales **5**
Cultural clubs **5**
Síndicatos **6**
Unions **6**
Otro (escribe el nombre) **88**
Other (write the name) **88**
No sabe **99**
Don't know **99**

66b. ¿Participas en estos grupos? **(groupar)**
Do you participate in these groups?
Si **1** [*Sigue a 66b1*]
Yes **1** **[***Go to 66b1***]**
No **0**
No **0**
No sabe **99**
Don't know **99**

66b1. Antes del problema de COVID, **¿**con qué frecuencia asistía en esos grupos? *Léeles las respuestas.* (**meetf1**)
Before the problem of COVID, how often did you attend these groups? *Read the answers.*
Más de una vez por semana **4**
More than once per week **4**
Una vez por semana **3**
Once a week **3**
Una o dos veces al mes **2**
Once or twice a month **2**
Una o dos veces al año **1**
Once or twice a year **1**
Nunca **0**
Never **0**
No sabe **99**
Don't know **99**

66b2. Ahora, dado la situación de COVID, ¿Como ha cambiado su participación esos grupos? **(covgroup)**
Now, given the COVID situation, how has your participation in these groups changed?
Igual **2**
The same **2**
Menor que antes **1**
Less than before **1**

Otro **88**
Other **88**
No sabe **99**
Don’t know **99**

Nivel socioeconómico
Socioeconomic level

Este es la ultima parte de la encuesta. Ahora me gustaría preguntarle acerca de las dificultades económicas.
This is the last part of the survey. Now I would like to ask you about financial hardship.

67. Piense en cuánto dinero gana usted y todos los demás en la casa al mes. Ahora, elija una de las siguientes respuestas: *Lee las respuestas disponibles.* **(finstress)**
    Think of how much money you and everyone else in the house makes per month. Now, choose one of the following answers: *Read available answers.*

    Los ingresos son lo suficientemente buenos para usted y puede ahorrar con ellos. **4**
    The income is good enough for you and you can save from it. **4**
    Los ingresos son lo suficientemente buenos para usted, por lo que no tiene mayores problemas. **3**
    The income is just good enough for you, so you do not have major problems. **3**
    Los ingresos no son suficientes para ti y estás estirado. **2**
    The income is not enough for you and you are stretched. **2**
    Los ingresos no son suficientes para usted y lo está pasando mal. **1**
    The income is not enough for you and you are having a hard time. **1**

68. ¿Tu casa tiene luz?
    Does your house have electricity? **(elec)**
    Si **1**
    Yes **1**
    No **0**
    No **0**
    No sabe **99**
    Don’t know **99**

69. ¿Tu casa tiene computadora?
    Does your house have a computer? **(comp)**
    Si **1**
    Yes **1**
    No **0**
    No **0**
    No sabe **99**
    Don’t know **99**

70. ¿Tu casa tiene internet?
    Does your house have the internet? **(int)**
    Si **1**
    Yes **1**
    No **0**
    No **0**
    No sabe **99**
    Don't know **99**

71. ¿Tu casa tiene televisión?
    Does your house have a television? **(tele)**
    Si **1**
    Yes **1**
    No **0**
    No **0**
    No sabe **99**
    Don't know **99**

72. ¿Alguien en su vivienda tiene carro proprio?
    Does anyone in your family own a car? **(car)**
    Si **1**
    Yes **1**
    No **0**
    No **0**
    No sabe **99**
    Don't know **99**

73. ¿La casa / apartamento / vivienda es propia o alquilada?
    Is the house rented or owned? **(ownhouse)**
    Propria **1**
    Owned **1**
    Alquilada **2**
    Rented **2**
    Otro **88**
    Other
    No sabe **99**
    Don't know

74. ¿Tiene seguro médico?
    Do you have health insurance? **(ins)**
    Si **1 [Sigue a la 74a]**
    Yes **1**
    No **0**
    No **0**
    No sabe **99**
    Don't know **99**

74a. ¿Es privado o publico su seguro? **(instype)**
Public **1**
Private **2**
Don't know **99**

75. *Observe los materiales de las paredes exteriores.*
Observe the materials of the exterior walls. **(wall)**
Madera **1**
Wood **1**
Metal / zinc **2**
Metal **2**
Block / cemento **3**
Brick / concrete **3**
Otro **88**
Other **88**

76. *Observe el material del techo.*
Observe the material of the roof. **(roof)**
Cana **1**
Straw/thatch **1**
Madera **2**
Wood **2**
Metal / zinc **3**
Metal **3**
Cemento **4**
Concrete **4**
Otro **88**
Other **88**

77. *Observe el material del piso interior.*
Observe the material of the interior floor. **(floor)**
Tierra **1**
Dirt/earth **1**
Madera **2**
Wood **2**
Cemento **3**
Concrete **3**
Cerámica **4**
Ceramic tiles **4**
Otro **88**
Other **88**

Hora de terminar **(ftime)**
Time

Gracias por tu tiempo. Sus respuestas ayudarán al programa contra la malaria a comprender mejor la situación en esta comunidad y otras similares, y con suerte desarrollar mejores programas para ayudarlo.

Thank you for your time. Your answers will help the malaria program better understand the situation in this community and others like it, and hopefully develop better programs to help you.
